# Supplementary material for: Spatial multi-omics identifies aggressive prostate cancer signatures highlighting pro-inflammatory chemokine activity in the tumor microenvironment
Source: Nat Commun. 2025 Nov 19;16:10160. doi: 10.1038/s41467-025-65161-9 (PMC12630738; doi:10.1038/s41467-025-65161-9)
Supplement: Supplementary file 1 — Supplementary Information [file 41467_2025_65161_MOESM1_ESM.pdf]

Supplementary Information to:

Spatial multi-omics identifies aggressive prostate cancer signatures highlighting pro-inflammatory chemokine activity in the tumor microenvironment

Sebastian Krossa[1,2,✉], Maria K. Andersen[1,3], Elise M. Sandholm[1,3], Maximilian Wess[1], Antti Kiviaho[4], Abhibhav Sharma[1,5], Sini Hakkola[4], Yangyang Hao[6], Mohammed Alshalalfa[6], Elai Davicioni[6], Trond Viset[7], Øystein Størkersen[7], R. Jeffrey Karnes[8], Daniel E. Spratt[9], Guro F. Giskeødegård[5], Matti Nykter[4,10], Morten B. Rye[3,11], Alfonso Urbanucci[4,12], and May-Britt Tessem[1,3,✉]

Affiliations

1. Department of Circulation and Medical Imaging, Norwegian University of Science and Technology
2. Central staff, St. Olavs Hospital HF, 7006, Trondheim, Norway
3. Clinic of Surgery, St. Olavs Hospital, Trondheim University Hospital, Trondheim, Norway
4. Prostate Cancer Research Center, Faculty of Medicine and Health Technology, Tampere University and TAYS Cancer Center, Tampere, Finland
5. HUNT Center for Molecular and Clinical Epidemiology, Department of Public Health and Nursing, Norwegian University of Science and Technology (NTNU), Trondheim, Norway
6. Veracyte, USA
7. Department of Pathology, St. Olavs Hospital, Trondheim University Hospital, Trondheim, Norway
8. Department of Urology, Mayo Clinic, Rochester, Minnesota, USA
9. UH Seidman Cancer Center, Case Western Reserve University, Cleveland, Ohio, USA.
10. Foundation for the Finnish Cancer Institute, Helsinki, Finland
11. Department of Clinical and Molecular Medicine, Norwegian University of Science and Technology
12. Department of Tumor Biology, Institute for Cancer Research, Oslo University Hospital, Oslo, Norway

✉Corresponding authors:

a. Sebastian Krossa: sebastian.krossa@ntnu.no
b. May-Britt Tessem: may-britt.tessem@ntnu.no

Content list

Supplementary Figure 1: Histopathology classes and signature identification flow chart.
Supplementary Figure 2: Spatial Transcriptomics data normalization by number of cells per spot.
Supplementary Figure 3: Spatial distribution of APC and CEG signature activity.
Supplementary Figure 4: Histopathology class composition of spots grouped by signature activity.
Supplementary Figure 5: Gene signature activity distribution in ST data.
Supplementary Figure 6: Spearman correlation of histopathology and cell types.
Supplementary Figure 7: Spearman correlation of all APC and CEG signature genes with cell types.
Supplementary Figure 8: Chemokine receptor expression detected in spatial and bulk transcriptomics data.
Supplementary Figure 9: Lipoteichoic acid (LTA, gram-positive bacteria) and lipopolysaccharides (LPS, gram-negative bacteria) staining results.
Supplementary Figure 10: APC signature score distribution in bulk samples from own cohort.
Supplementary Figure 11: APC signature score distribution in bulk samples from own cohort.
Supplementary Table 1: Clinical data of all 37 patients with prostate tissue transcriptomics profiling in this study.
Supplementary Table 2: List of gene sets used for ssGSEA analysis.
Supplementary Table 3: Data sets used for analysis in public data.
Supplementary Table 4: Patient characteristics table META855 cohort by APC signature tertiles.
Supplementary Table 5: Patient characteristics table META855 cohort by CEG signature tertiles.
Supplementary Table 6: Univariable and multivariable analysis for biochemical recurrence using META855 cohort for APC signature tertiles.
Supplementary Table 7: Univariable and multivariable analysis for metastasis using META855 cohort for APC signature tertiles.

Supplementary Table 8: Univariable and multivariable analysis for biochemical recurrence using META855 cohort for CEG signature tertiles.

Supplementary Table 9: Univariable and multivariable analysis for metastasis using META855 cohort for CEG signature tertiles.

Supplementary Figures

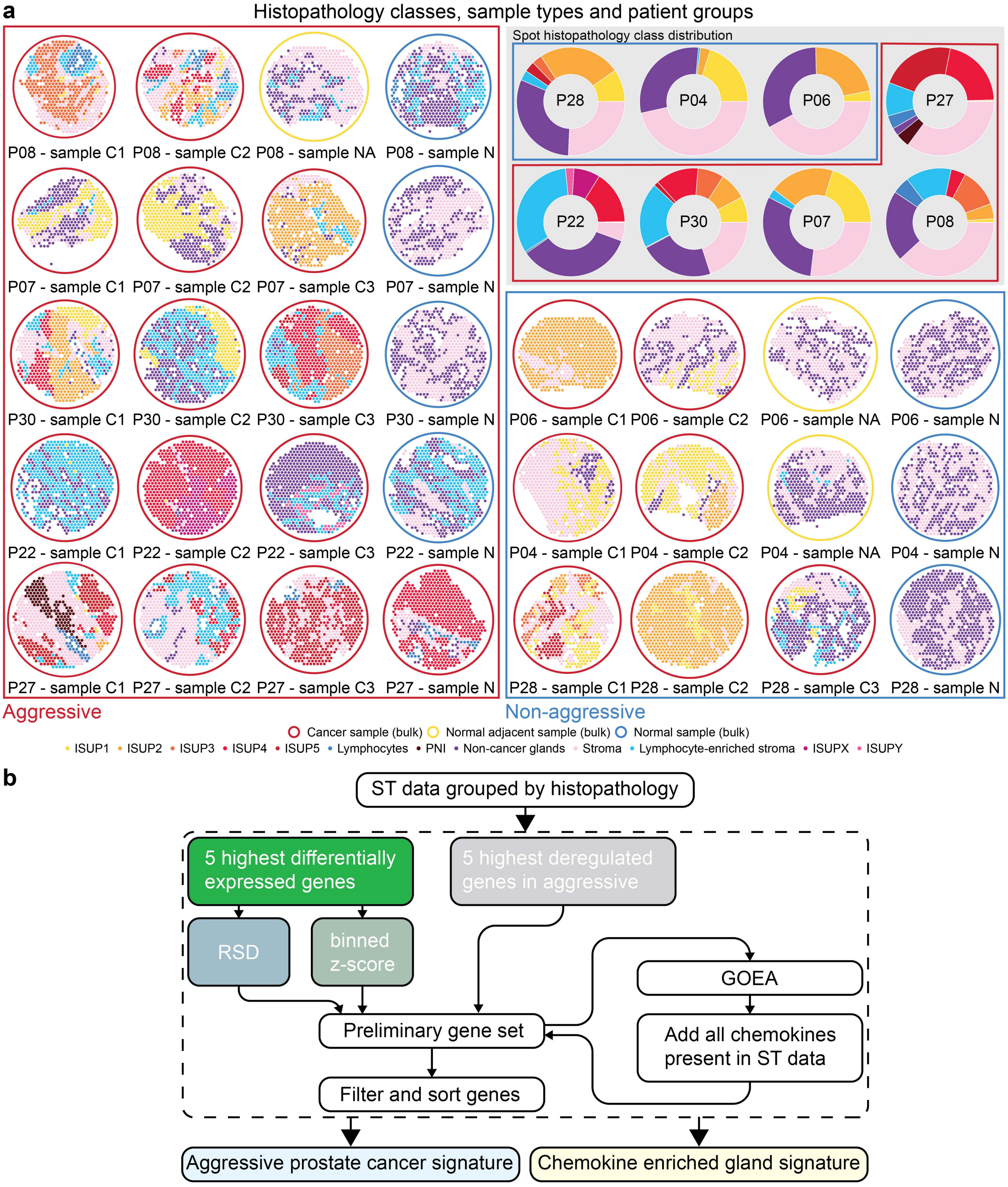

Supplementary Figure 1. ***Histopathology classes and signature identification flow chart.*** *(a) Histopathology classes visualized for all spots (n=19854) of all samples (n=32) grouped by patient class (left, red box = aggressive PCa, right, blue box = non-aggressive PCa), sorted by patients (rows) and sample type (columns: C = cancer, NA = normal adjacent, N = normal). Insert top right is showing the histopathology class distribution for each patient grouped by class (blue = non-aggressive PCa, red = aggressive PCa). (b) Flow chart illustrating the algorithm used to generate the two signatures: Aggressive prostate cancer (APC) and Chemokine enriched gland (CEG).*

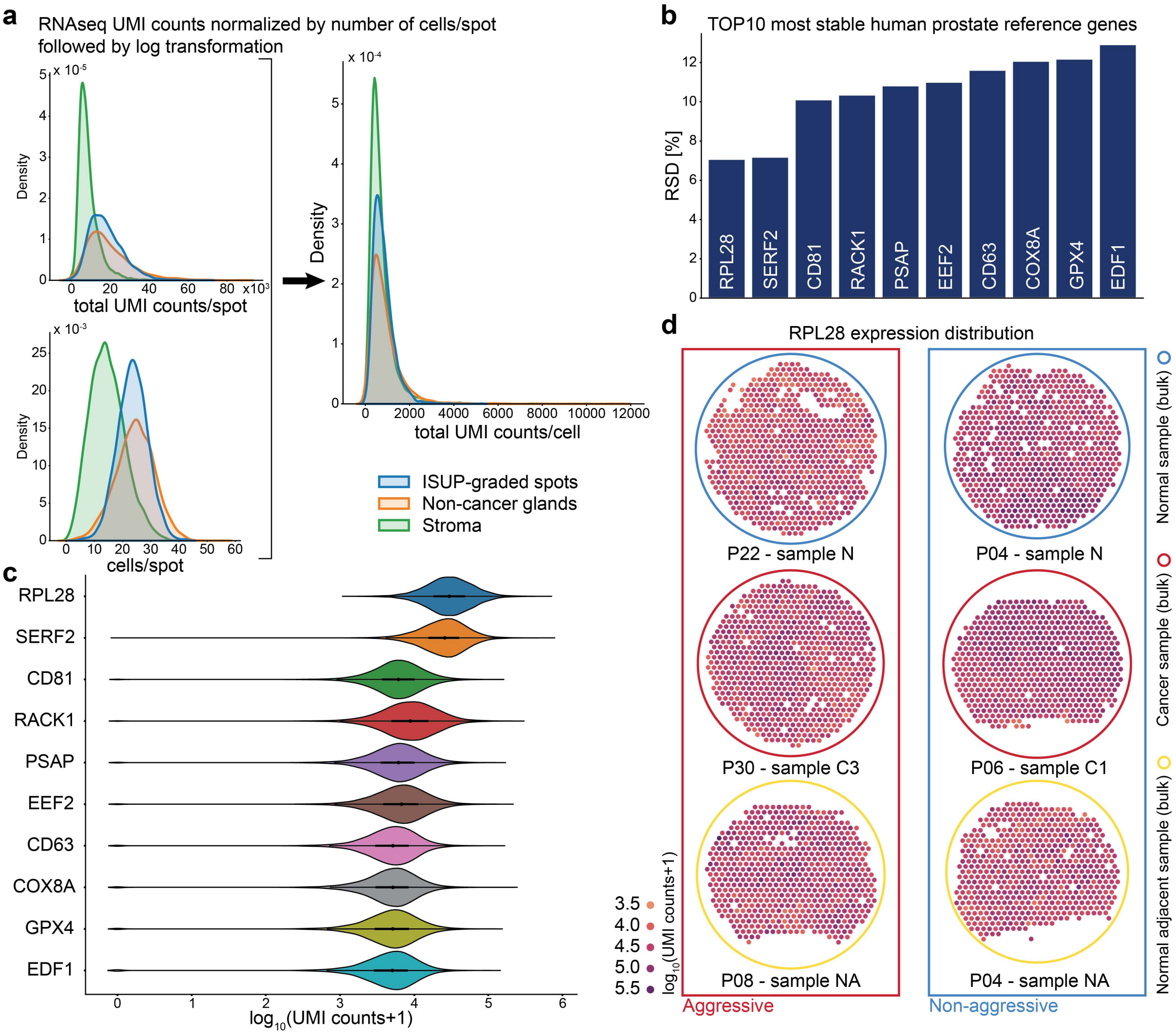

Supplementary Figure 2 ***Spatial Transcriptomics data normalization by number of cells per spot****. (a) The total UMI reads/spot and number of cells/spot were increased in cancer and NCG spots as compared to stroma spots. Scaling the reads by number of cells per spot results in spot-type independent total UMI reads distributions (shown as). (b) The relative standard deviation (RSD) of 10 select reference genes for the human prostate were all below 13% after normalization by cells/spot and showed comparable intensity (c) shown as violin plots of all ST spots (n=19854) and spatial (d) distributions shown exemplary for 6 samples from 5 patients after log10 variance stabilization over all samples.*

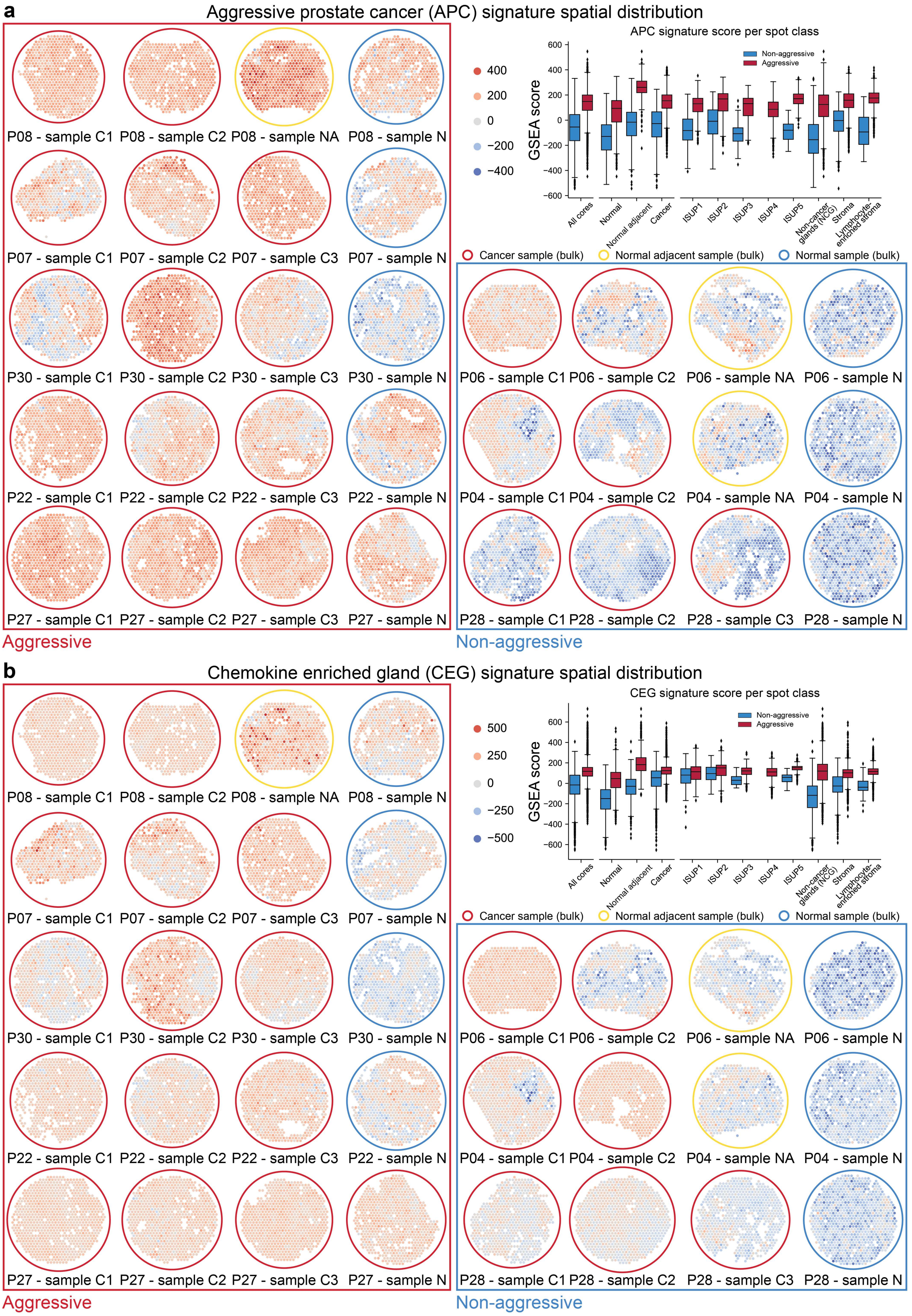

Supplementary Figure 3 ***Spatial distribution of aggressive prostate cancer (APC) and chemokine enriched gland (CEG) signature activity****. (a) Distribution of the APC and (b) CEG score in all ST samples grouped by patient class (left, red box = aggressive PCa, right, blue box = non-aggressive PCa), sorted by patients (rows) and sample type (columns: cancer, adjacent*

normal, normal). Inserts show respective score distributions as box-and-whisker plots (box spans interquartile range (IQR), centerline indicates median, whisker extend 1.5 IQR from first and third quartile, observations beyond shown as individual points) separated by patient class (blue = non-aggressive PCa, red = aggressive PCa) for all cores/samples and grouped by core type (normal, normal adjacent, cancer), and histopathology classes (ISUP1-5, NCG, stroma, lymphocyte-enriched stroma)

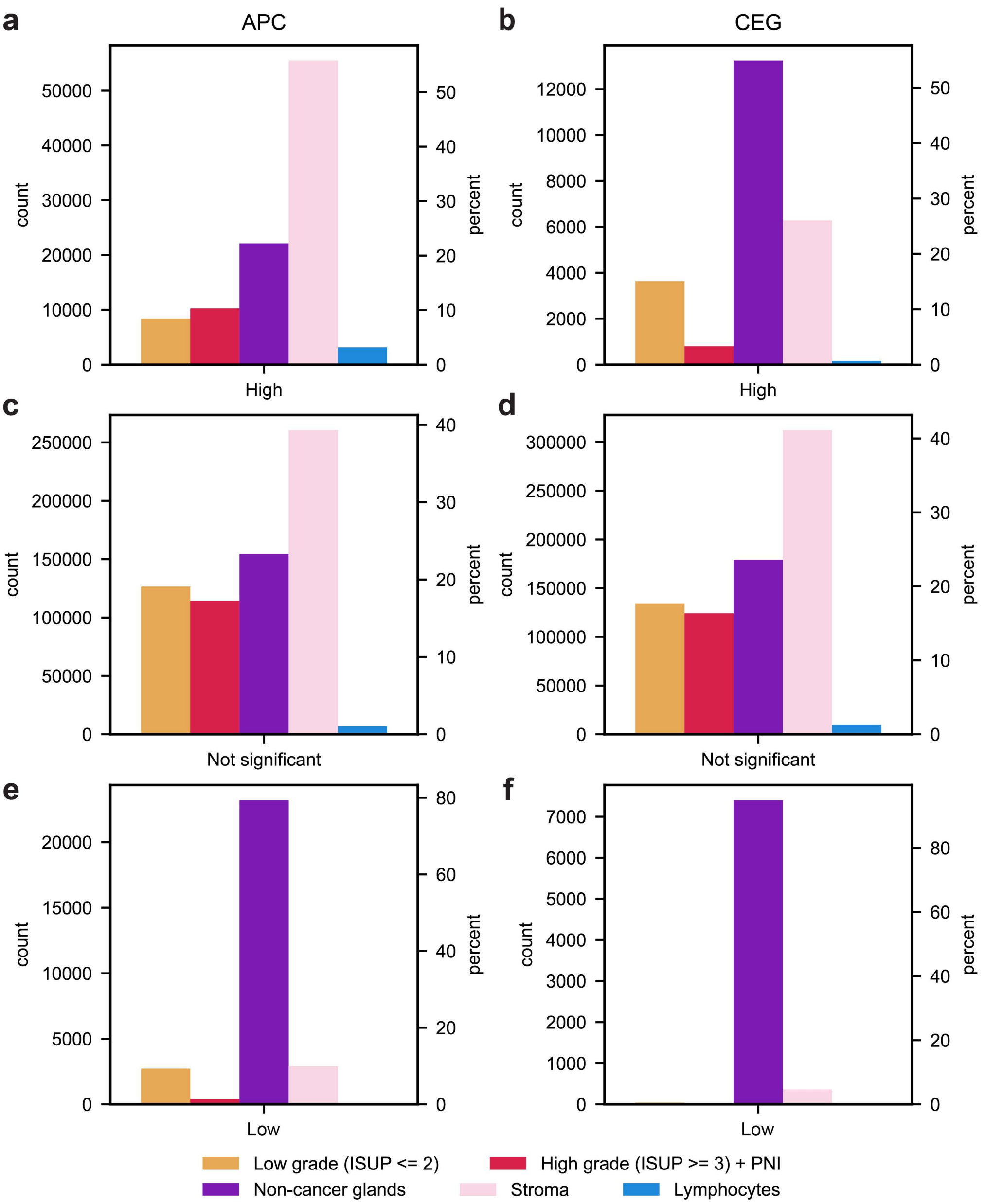

Supplementary Figure 4 ***Histopathology class composition of spots grouped by signature activity.*** *The compositions are shown for (a) high, (c) not significant, (d) low APC and (b) high, (d) not significant, (f) low CEG signature scoring spots as spot counts and percentage of all spots of the respective signature scoring group. Cancer spots were grouped into low grade (all spots classified as ISUP1-2) and high grade (all spots classified as ISUP3–5 and PNI).*

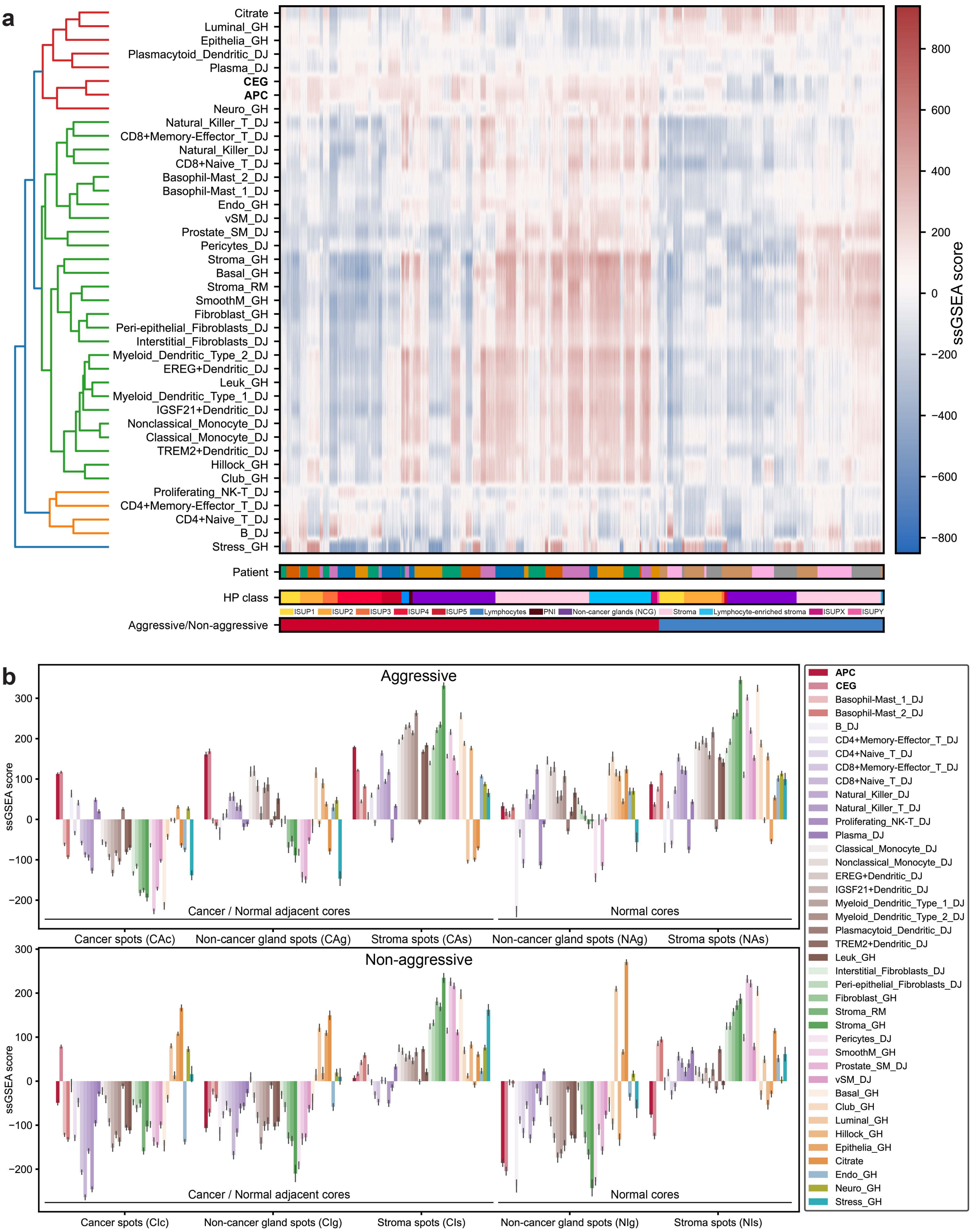

Supplementary Figure 5. ***Gene signature activity distribution in ST data.*** *(a) Signature scores are visualized for each spot by color, grouped as indicated by color bars in the 3 rows at the bottom by patient class (blue = non-aggressive PCa, red = aggressive PCa), histopathology (HP), and patients. Gene sets/signatures were clustered according to distribution similarity to enhance readability. (b) Mean (+/- standard deviation) activity of all prostate-related gene sets, cell type specific gene set, APC, and CEG signature of ST data spots group according to sample type (cancer/normal adjacent or normal), patient class (aggressive/non-aggressive PCa), and spot class (Non-cancer gland [NCG], cancer, stroma) derived from ST data of 32 samples (non-aggressive n=12, aggressive n=20) from 8 (non-aggressive n=3, aggressive n=5) PCa patients, ST spots per group: CAc n=4272, CAg n=1623, CAs n=3754, CIc n=2218, CIg n=1181, CIs n=2004, NAg n=1101, NAs n=1367, NIg n=1107, NIs n=863.*

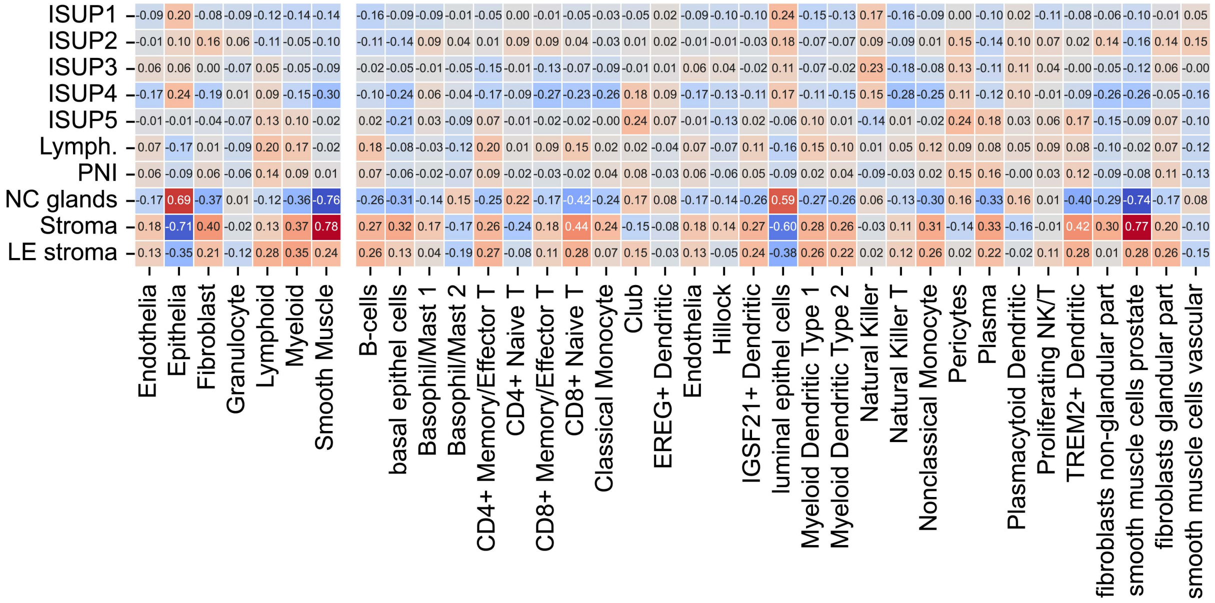

Supplementary Figure 6 ***Spearman correlation of histopathology and cell types****. Correlation coefficients are given as numbers and additionally visualized by color (blue = negative, red = positive correlation). Correlation coefficients were calculated using the raw histopathology class fractions per spot (see Methods) and cell type fractions obtained by spot deconvolution using ST data (spots n=19854) of 32 samples (non-aggressive n=12, aggressive n=20) from 8 (non-aggressive n=3, aggressive n=5) PCa patients.*

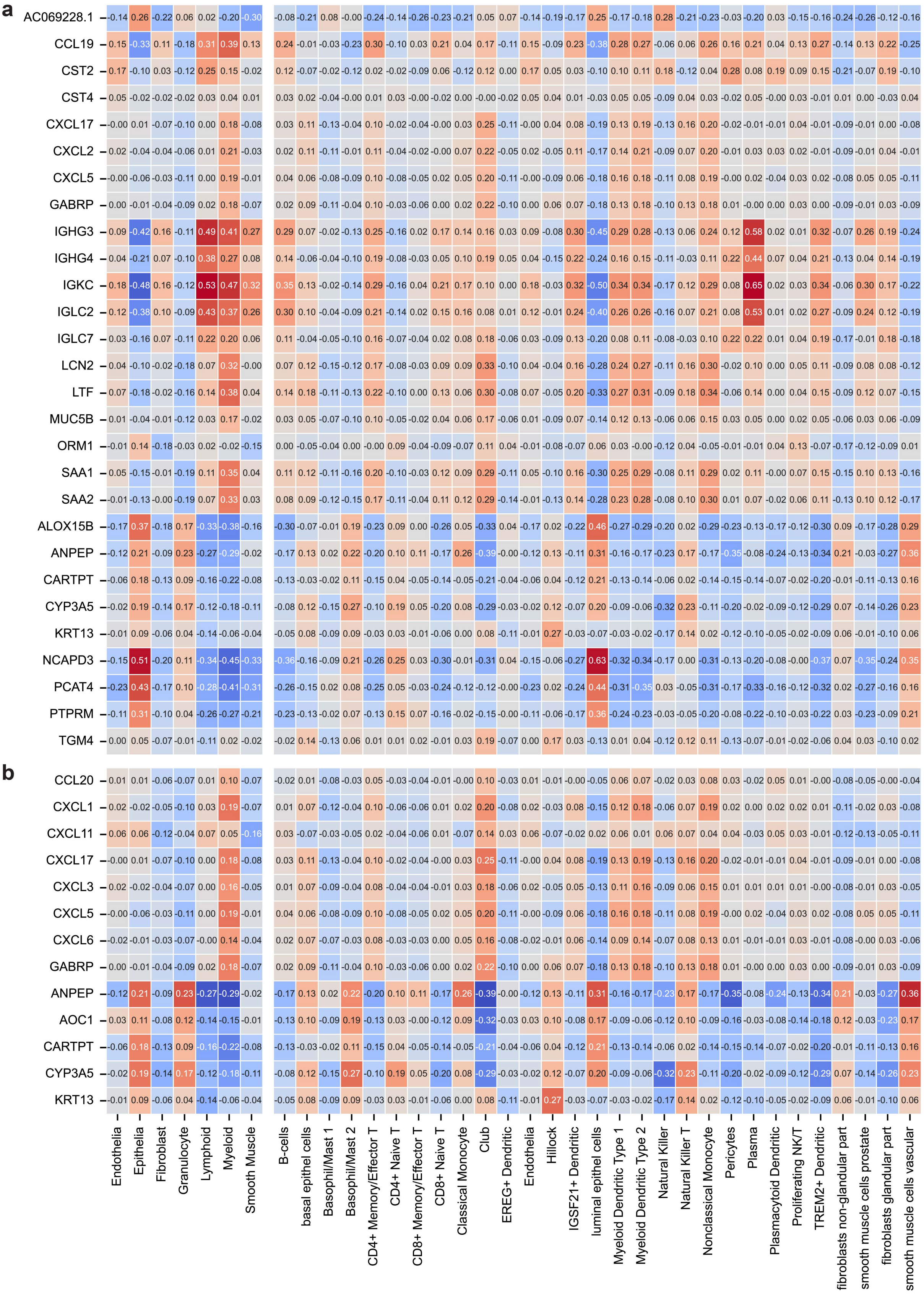

Supplementary Figure 7 ***Spearman correlation of all APC (a) and CEG (b) signature genes with cell types****. Correlation coefficients are given as numbers and additionally visualized by color (blue = negative, red = positive correlation). Correlation coefficients were calculated using the normalized reads per spot (see Methods) and cell type fractions obtained by spot*

deconvolution using ST data (spots n=19854) of 32 samples (non-aggressive n=12, aggressive n=20) from 8 (non-aggressive n=3, aggressive n=5) PCa patients.

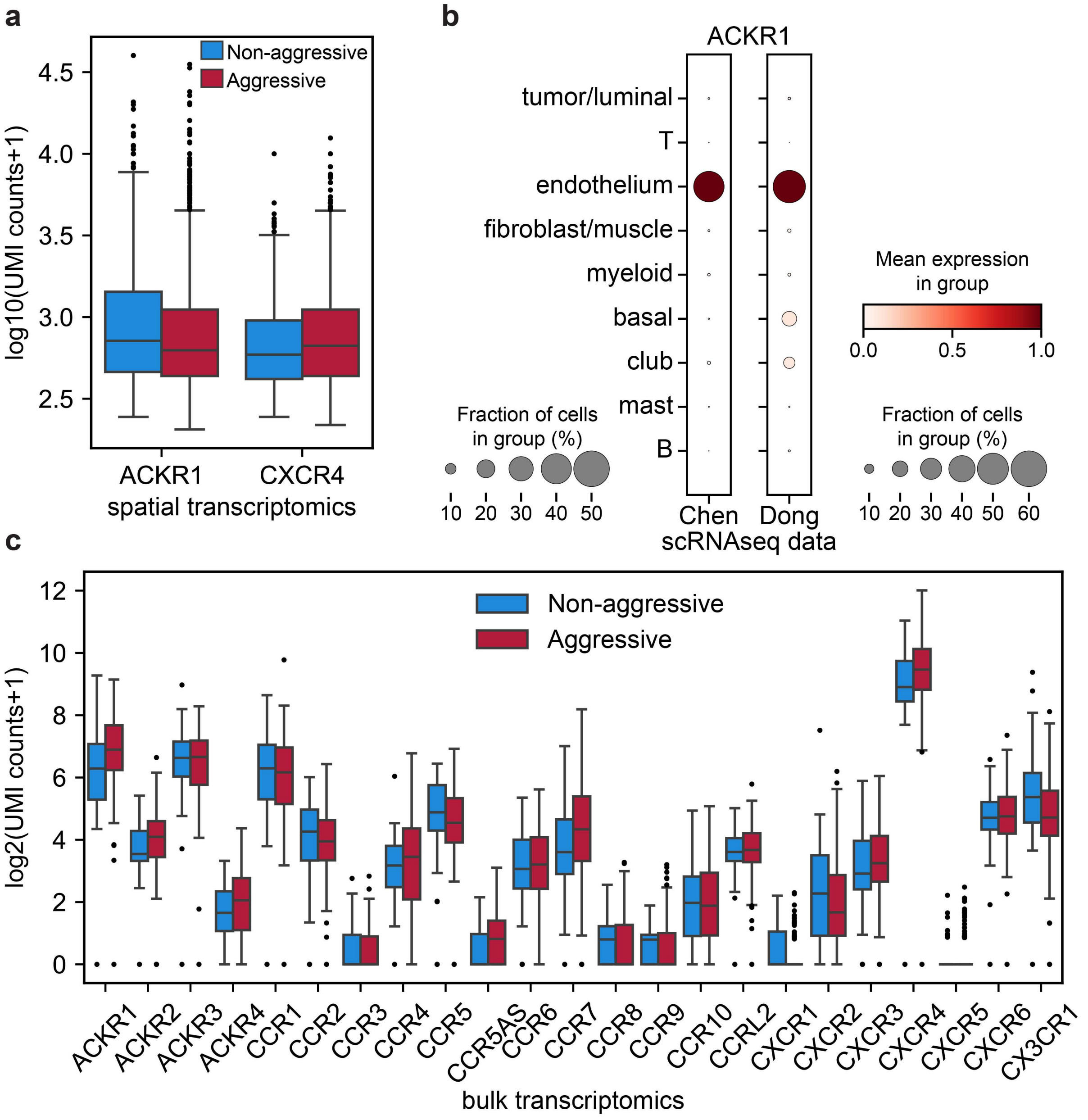

Supplementary Figure 8 ***Chemokine receptor expression detected in spatial, single cell, and bulk transcriptomics data.*** *Logarithm-transformed normalized expressions obtained by (a) spatial transcriptomics of 32 samples (non-aggressive n=12, aggressive n=20) from 8 (non-aggressive n=3, aggressive n=5) PCa patients displaying spots with UMI counts > 0 of the respective genes (ACKR1 non-aggressive n=1394, aggressive n=2557, CXCR4 non-aggressive n=1416, aggressive n=5090 spots) and (c) bulk transcriptomics of 174 samples (non-aggressive n=48, aggressive n=126) from 37 PCa patients (non-aggressive n=10, aggressive n=37) are shown as box-and-whisker plots (box spans interquartile range (IQR), centerline indicates median, whisker extend 1.5 IQR from first and third quartile, observations beyond shown as individual points) separated by patient class (blue = non-aggressive, red = aggressive PCa). (b) Expression of ACKR1 receptor in different cell types derived from scRNAseq data from prostate tumors (Cheng) [27] and CRPC needle biopsies (Dong) [28] visualized as dot-plots representing the fraction of cells in each group by diameter and the normalized mean expression of ACKR1 by color.*

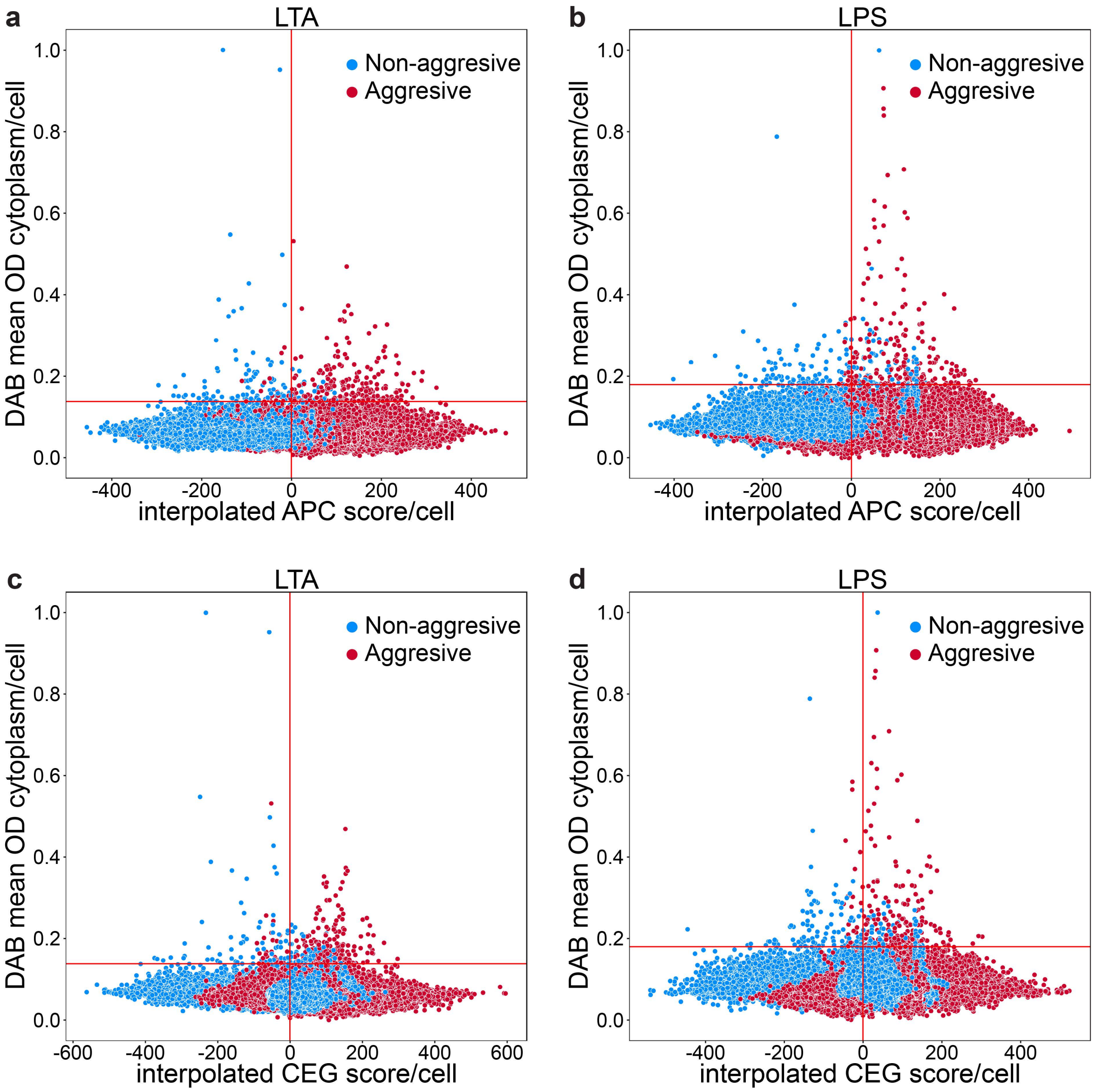

Supplementary Figure 9. ***Lipoteichoic acid (LTA, gram-positive bacteria) and lipopolysaccharides (LPS, gram-negative bacteria) staining.*** *LTA (a, c) and LPS (b, d) cells computationally extracted from stained tissue were assigned mean DAB optical density (OD) values min-max normalized between 0 and 1 based on the staining intensity and an interpolated APC (a, b) and CEG (c, d) signature score (see Methods for details). Every cell detected is visualized as a point in the scatter plots grouped by patient class (blue = non-aggressive, red = aggressive PCa). Horizontal red lines indicate positive DAB staining cutoff.*

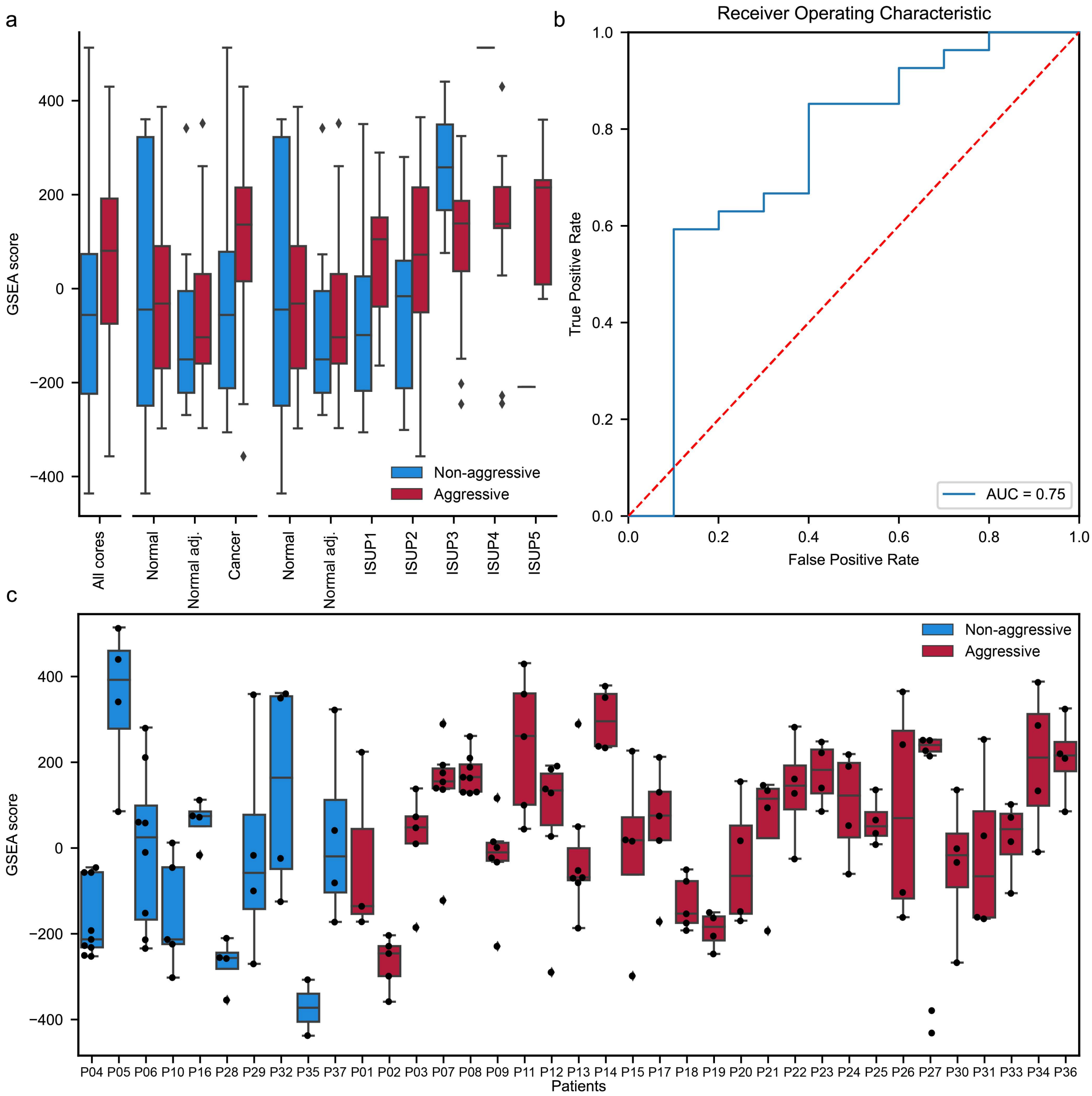

Supplementary Figure 10. ***APC signature score distribution in bulk samples from own cohort.*** *(a) Distributions are shown as box-and-whisker plots separated by patient class (blue = non-aggressive, red = aggressive PCa) and shown for all cores (aggressive n=126, non-aggressive n=48) from all patients (aggressive n=27, non-aggressive n=10), grouped by core/sample type (normal n=27+9 [aggressive + non-aggressive], normal adjacent n=14+11, cancer n=27+9), and sample/core type and ISUP grade (normal n=27+9 [aggressive + non-aggressive], normal adjacent n=14+11, ISUP1 n=10+15, IUSP2 n=27+9, ISUP3 n=19+2, ISUP4 n=18+1, ISUP5 n=11+1). (b) Receiver operating characteristics (ROC) of the APC signature in cancer bulk samples (aggressive n=85, non-aggressive n=28) resulted in an area under the curve (AUC) of 0.75. (c) Signature score distribution shown as box-and-whisker plots overlayed with each observation (black circles) of all samples per patient class (blue = non-aggressive, red = aggressive PCa). For all box-and-whisker plots box spans interquartile range (IQR), centerline indicates median, whisker extend 1.5 IQR from first and third quartile, observations beyond shown as individual points.*

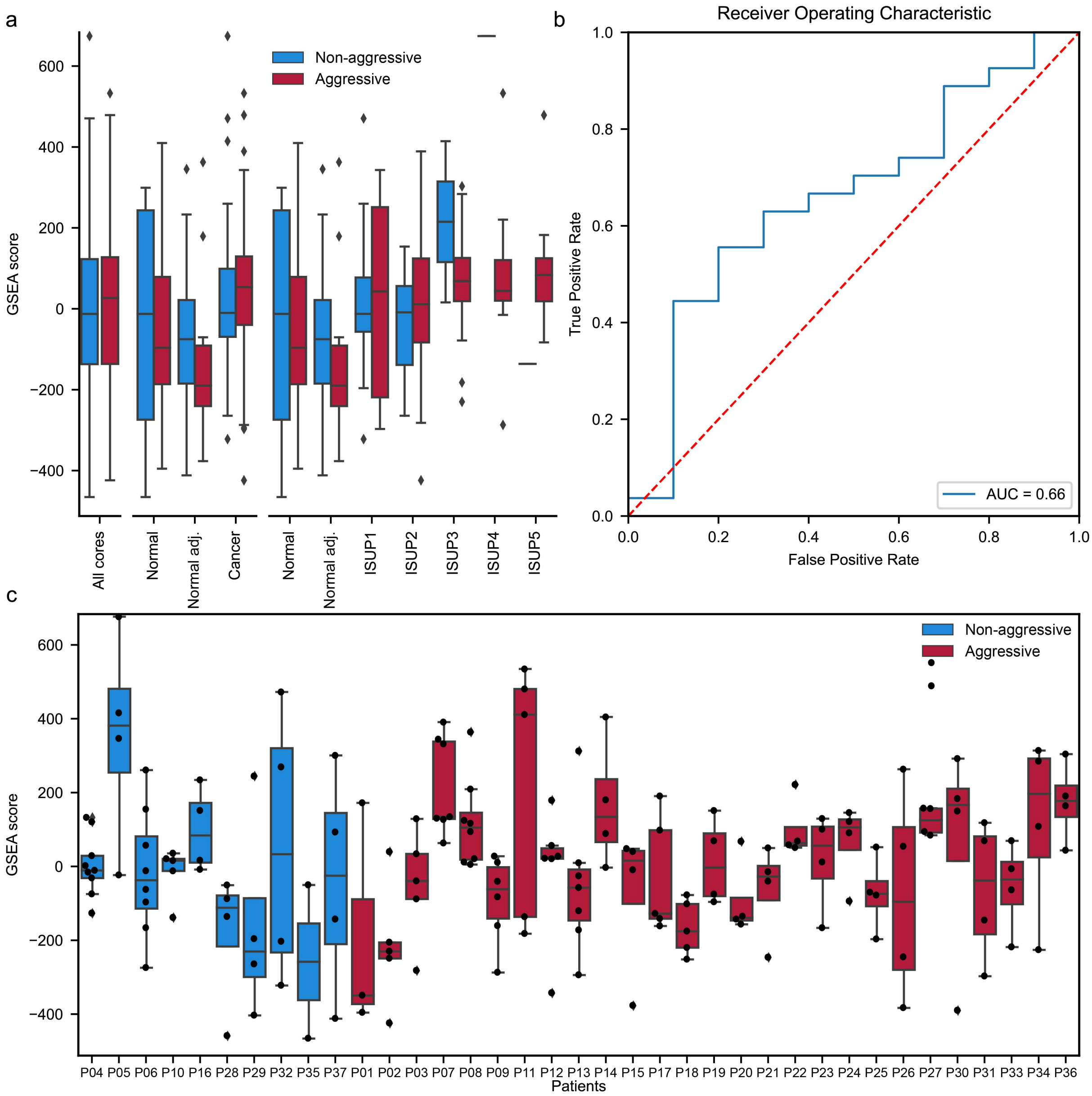

Supplementary Figure 11. ***CEG signature score distribution in bulk samples from own cohort.*** *(a) Distributions are shown as box-and-whisker plots separated by patient class (blue = non-aggressive, red = aggressive PCa) and shown for all cores (aggressive n=126, non-aggressive n=48) from all patients (aggressive n=27, non-aggressive n=10), grouped by core/sample type (normal n=27+9 [aggressive + non-aggressive], normal adjacent n=14+11, cancer n=27+9), and sample/core type and ISUP grade (normal n=27+9 [aggressive + non-aggressive], normal adjacent n=14+11, ISUP1 n=10+15, IUSP2 n=27+9, ISUP3 n=19+2, ISUP4 n=18+1, ISUP5 n=11+1). (b) Receiver operating characteristics (ROC) of the CEG signature in bulk samples (aggressive n=85, non-aggressive n=28) resulted in an area under the curve (AUC) of 0.66. (c) Signature score distribution shown as box-and-whisker plots overlayed with each observation (black circles) of all samples per patient class (blue = non-aggressive, red = aggressive PCa). For all box-and-whisker plots box spans interquartile range (IQR), centerline indicates median, whisker extend 1.5 IQR from first and third quartile, observations beyond shown as individual points.*

Supplementary Tables

Supplementary Table 1 Clinical data of all 37 patients with prostate tissue transcriptomics profiling in this study. Patients with both spatial transcriptomics and bulk transcriptomics profiling are marked in bold. Persistent PCa indicated by 0 month and relapse-free PCa indicated by – in Time to relapse column.

ID	Status	Time to relapse (months)	Follow up time w. PSA	Total follow up time	Age in range at operation	Mean preoperative PSA	Preoperative Grade group	Clinical T-stage	EAU risk group for biochemical recurrence**	Operative Grade group	Post op. T-stage
P01	Aggressive	32	78	78	60-69	7.3	5	T3	H	4	T3a
P02	Aggressive	0	102	102	60-69	9	3	T1c	I	3	T2c
P03	Aggressive	0	62	62	50-59	47.9	3	T3b	H	5	T3b
**P04**	**Non-aggressive**	**–**	**135**	**135**	**60-69**	**10**	**2**	**T2**	**I**	**2**	**T2c**
P05	Non-aggressive	–	99	117	60-69	5.4	2	T2	I	2	T2c
**P06**	**Non-aggressive**	**–**	**105**	**168**	**50-59**	**9.6**	**2**	**T1c**	**I**	**2**	**NA***
**P07**	**Aggressive**	**37^{m-90}**	**107**	**107**	**50-59**	**12.8**	**3**	**T2**	**I**	**2**	**T3b**
**P08**	**Aggressive**	**0^{m-22}**	**78**	**78**	**50-59**	**11**	**5**	**T2b**	**H**	**5**	**T3a**
P09	Aggressive	0	62	62	50-59	26.4	5	T3b	H	5	T3b
P10	Non-aggressive	–	59	183	50-59	16.4	2	T2/3	H	2	T2c
P11	Aggressive	14	79	79	50-59	18.4	2	T2a	I	3	T3b
P12	Aggressive	114	141	141	60-69	9.8	3	T3a	H	3	T2c
P13	Aggressive	11	56	56	60-69	19	1	T1c	I	2	T2c
P14	Aggressive	0	2	2	60-69	6.2	5	T3b	H	5	T3b
P15	Aggressive	1	33	33	50-59	60	5	T3b	H	4	T3b
P16	Non-aggressive	–	184	187	60-69	6.4	2	T2	I	2	T2c
P17	Aggressive	19	70	70	60-69	9.5	2	NA*	I	2	T3a
P18	Aggressive	44	50	50	60-69	5.9	2	T2c	H	2	T2c
P19	Aggressive	0	93	93	60-69	4.6	4	NA*	H	2	T2c
P20	Aggressive	15	102	102	50-59	13.9	2	T2	I	2	T2c
P21	Aggressive	0	39	39	60-69	11.4	5	T3a	H	5	T3b
**P22**	**Aggressive**	**0**	**34**	**34**	50-59	**32.5**	**3**	**T3a**	**H**	**4**	**T3a**
P23	Aggressive	45	53	53	60-69	11.6	3	T2c	H	3	T3a
P24	Aggressive	25	66	66	60-69	4.8	3	T2b	I	3	T3a
P25	Aggressive	2	48	48	60-69	15.1	4	T2b	H	4	T3b
P26	Aggressive	107	125	125	60-69	7.7	2	NA*	I	3	T2c
**P27**	**Aggressive**	**0**	**44**	**44**	**70-79**	**16.3**	**5**	**T3b**	**H**	**5**	**T3b**
**P28**	**Non-aggressive**	**–**	**140**	**166**	**60-69**	**22.3**	**2**	**T2**	**H**	**3**	**T2c**
P29	Non-aggressive	–	168	178	50-59	10.2	2	T3	H	2	T2c
**P30**	**Aggressive**	**0**	**22**	**22**	**50-59**	**45.9**	**4**	**T2c**	**H**	**3**	**T2c**
P31	Aggressive	45	45	45	50-59	52.5	2	T3	H	2	T3b
P32	Non-aggressive	–	99	170	50-59	11.5	2	T1c	I	2	T2c
P33	Aggressive	7	44	44	70-79	7.3	2	T2b	I	3	T3b
P34	Aggressive	38	83	83	60-69	8.4	2	T2c/T3	H	3	T2c
P35	Non-aggressive	–	20	176	50-59	5.6	2	T1c	I	2	T2c
P36	Aggressive	0	30	30	70-79	29	3	T3a	H	3	T3b
P37	Non-aggressive	–	157	188	50-59	10.2	5	T3	H	3	T2c

$^{m-N}$ *Aggressive PCa with confirmed metastasis after N month*

** Information was not available.*

*** EAU intermediate = I, high = H risk group for biochemical recurrence according to EAU based on mean preoperative.*

Supplementary Table 2: List of gene sets used for ssGSEA analysis. References below this Table.

Gene set name	Number of genes	Genes in ST data	Source	Comment
B_DJ	75	54	Joseph, 2021, JrnPath [1]	
Basophil-Mast_1_DJ	75	43	Joseph, 2021, JrnPath	
Basophil-Mast_2_DJ	75	42	Joseph, 2021, JrnPath	
CD4+Memory-Effector_T_DJ	75	45	Joseph, 2021, JrnPath	
CD4+Naive_T_DJ	75	37	Joseph, 2021, JrnPath	CD4+ in Azimuth
CD8+Memory-Effector_T_DJ	75	26	Joseph, 2021, JrnPath	
CD8+Naive_T_DJ	75	28	Joseph, 2021, JrnPath	CD8+ in Azimuth
Classical_Monocyte_DJ	75	32	Joseph, 2021, JrnPath	
EREG+Dendritic_DJ	75	41	Joseph, 2021, JrnPath	
IGSF21+Dendritic_DJ	75	41	Joseph, 2021, JrnPath	
Interstitial_Fibroblasts_DJ	75	38	Joseph, 2021, JrnPath	
Myeloid_Dendritic_Type_1_DJ	75	29	Joseph, 2021, JrnPath	
Myeloid_Dendritic_Type_2_DJ	75	35	Joseph, 2021, JrnPath	
Natural_Killer_DJ	75	21	Joseph, 2021, JrnPath	
Natural_Killer_T_DJ	75	22	Joseph, 2021, JrnPath	
Nonclassical_Monocyte_DJ	75	33	Joseph, 2021, JrnPath	
Peri-epithelial_Fibroblasts_DJ	75	38	Joseph, 2021, JrnPath	
Pericytes_DJ	75	45	Joseph, 2021, JrnPath	
Plasma_DJ	75	42	Joseph, 2021, JrnPath	
Plasmacytoid_Dendritic_DJ	75	24	Joseph, 2021, JrnPath	
Proliferating_NK-T_DJ	75	46	Joseph, 2021, JrnPath	
Prostate_SM_DJ	75	61	Joseph, 2021, JrnPath	
TREM2+Dendritic_DJ	75	61	Joseph, 2021, JrnPath	
vSM_DJ	75	38	Joseph, 2021, JrnPath	
Basal_GH	30	18	Henry, 2018, CellRep [2]	
Club_GH	69	56	Henry, 2018, CellRep	
Endo_GH	111	47	Henry, 2018, CellRep	
Epithelia_GH	166	56	Henry, 2018, CellRep	
Fibroblast_GH	124	61	Henry, 2018, CellRep	
Hillock_GH	54	30	Henry, 2018, CellRep	
Leuk_GH	135	65	Henry, 2018, CellRep	
Luminal_GH	71	63	Henry, 2018, CellRep	
Neuro_GH	560	55	Henry, 2018, CellRep	
SmoothM_GH	99	61	Henry, 2018, CellRep	
Stress_GH	13	13	Henry, 2018, CellRep	
Stroma_GH	905	96	Henry, 2018, CellRep	
Stroma_RM	150	42	Tessem, 2016, PLOSOne [3]	
Citrate	150	109	Rye, 2022, iScience [4]	
GPS	12	*na*	Na, 2016, AsJrnAndr [5]	
Decipher	19	*na*	Na, 2016, AsJrnAndr	
CCP	31	*na*	Na, 2016, AsJrnAndr	
RA	26	26	This Study	
CEG	12	12	This Study	

1. Joseph DB, Henry GH, Malewska A, Reese JC, Mauck RJ, Gahan JC, Hutchinson RC, Malladi VS, Roehrborn CG, Vezina CM et al: Single-cell analysis of mouse and human prostate reveals novel fibroblasts with specialized distribution and microenvironment interactions. J Pathol 2021, 255(2):141-154.
2. Henry GH, Malewska A, Joseph DB, Malladi VS, Lee J, Torrealba J, Mauck RJ, Gahan JC, Raj GV, Roehrborn CG et al: A Cellular Anatomy of the Normal Adult Human Prostate and Prostatic Urethra. Cell reports 2018, 25(12):3530-3542 e3535.
3. Tessem MB, Bertilsson H, Angelsen A, Bathen TF, Drablos F, Rye MB: A Balanced Tissue Composition Reveals New Metabolic and Gene Expression Markers in Prostate Cancer. PLoS One 2016, 11(4):e0153727.
4. Rye MB, Krossa S, Hall M, van Mourik C, Bathen TF, Drablos F, Tessem MB, Bertilsson H: The genes controlling normal function of citrate and spermine secretion are lost in aggressive prostate cancer and prostate model systems. Iscience 2022, 25(6):104451.
5. Na R, Wu YS, Ding Q, Xu JF: Clinically available RNA profiling tests of prostate tumors: utility and comparison. Asian journal of andrology 2016, 18(4):575-579.

Supplementary Table 3: Data sets used for analysis in public data. References below this Table.

ID	Dataset Abbreviation	Description	Data set source	Reference
1	Bertilsson	156 prostate tissue samples (116 cancer and 40 normal)	Array Express: E-MTAB-1041	[1]
2	Wang	136 prostate tissue samples 65 cancer and 71 normal	GEO: GSE8218	[2, 3]
3	Taylor	160 prostate tissue samples (131 cancer and 29 normal)	GEO: GSE21034	[4]
4	Sboner	281 prostate cancer samples	GEO: GSE16560	[5]
5	Erho	545 prostate cancer samples	GEO: GSE46691	[6]
6	TCGA-PRAD	549 prostate tissue samples (497 cancer and 52 normal)	https://portal.gdc.cancer.gov/repository	https://www.cancer.gov/tcga
7	CMBR (Cambridge)	186 prostate tissue samples (112 cancer and 74 normal)	GEO: GSE70768	[7]
8	STCK (Stockholm)	94 prostate cancer samples	GEO: GSE70769	[7]
9	Mortensen	50 prostate tissue samples (36 cancer and 14 normal) Laser dissected tissue	GEO: GSE46602	[8]
10	Kuner	98 prostate tissue samples (59 cancer and 39 normal)	GEO: GSE32571	[9]
11	Loda	Laser Dissected tissue from 188 normal and cancer samples	GEO: GSE97284	[10]
12	GTEx-Prostate	245 prostate normal samples	https://gtexportal.org/home/datasets	https://gtexportal.org/home/

1. Bertilsson H, Tessem MB, Flatberg A, Viset T, Gribbestad I, Angelsen A, Halgunset J: Changes in Gene Transcription Underlying the Aberrant Citrate and Choline Metabolism in Human Prostate Cancer Samples. Clin Cancer Res 2012, 18(12):3261-3269.
2. Wang Y, Xia XQ, Jia Z, Sawyers A, Yao H, Wang-Rodriquez J, Mercola D, McClelland M: In silico estimates of tissue components in surgical samples based on expression profiling data. Cancer Res 2010, 70(16):6448-6455.
3. Jia Z, Wang Y, Sawyers A, Yao H, Rahmatpanah F, Xia XQ, Xu Q, Pio R, Turan T, Koziol JA et al: Diagnosis of prostate cancer using differentially expressed genes in stroma. Cancer Res 2011, 71(7):2476-2487.
4. Taylor BS, Schultz N, Hieronymus H, Gopalan A, Xiao Y, Carver BS, Arora VK, Kaushik P, Cerami E, Reva B et al: Integrative genomic profiling of human prostate cancer. Cancer cell 2010, 18(1):11-22.
5. Sboner A, Demichelis F, Calza S, Pawitan Y, Setlur SR, Hoshida Y, Perner S, Adami HO, Fall K, Mucci LA et al: Molecular sampling of prostate cancer: a dilemma for predicting disease progression. BMC medical genomics 2010, 3:8.
6. Erho N, Crisan A, Vergara IA, Mitra AP, Ghadessi M, Buerki C, Bergstralh EJ, Kollmeyer T, Fink S, Haddad Z et al: Discovery and Validation of a Prostate Cancer Genomic Classifier that Predicts Early Metastasis Following Radical Prostatectomy. PLoS One 2013, 8(6):e66855.
7. Ross-Adams H, Lamb AD, Dunning MJ, Halim S, Lindberg J, Massie CM, Egevad LA, Russell R, Ramos-Montoya A, Vowler SL et al: Integration of copy number and transcriptomics provides risk stratification in prostate cancer: A discovery and validation cohort study. Ebiomedicine 2015, 2(9):1133-1144.
8. Mortensen MM, Hoyer S, Lynnerup AS, Orntoft TF, Sorensen KD, Borre M, Dyrskjot L: Expression profiling of prostate cancer tissue delineates genes associated with recurrence after prostatectomy. Scientific reports 2015, 5:16018.
9. Kuner R, Falth M, Pressinotti NC, Brase JC, Puig SB, Metzger J, Gade S, Schafer G, Bartsch G, Steiner E et al: The maternal embryonic leucine zipper kinase (MELK) is upregulated in high-grade prostate cancer. J Mol Med 2013, 91(2):237-248.
10. Tyekucheva S, Bowden M, Bango C, Giunchi F, Huang Y, Zhou C, Bondi A, Lis R, Van Hemelrijck M, Andren O et al: Stromal and epithelial transcriptional map of initiation progression and metastatic potential of human prostate cancer. Nature communications 2017, 8(1):420.

Supplementary Table 4: Patient characteristics table META855 cohort by APC signature tertiles. Statistical tests used to obtain two-sided p-values (no multiple testing adjustment) are given below the table. Full data used to generate this table, and exact p-values provided in source data file for supplement.

Variables	Overall	APC-tertiles			P-value
		0%-33%	33%-67%	67%-100%	
**Total, No. (%)**	855 (100.0)	282 (33.0)	291 (34.0)	282 (33.0)	
**Race, No. (%)**					
White	731 (85.8)	244 (87.1)	247 (84.9)	240 (85.4)	0.915[b]
Black	104 (12.2)	30 (10.7)	38 (13.1)	36 (12.8)	
Other	17 (2.0)	6 (2.1)	6 (2.1)	5 (1.8)	
**Age, years [median (Q1, Q3)]**	60 (55, 65)	60 (55, 65)	60 (54, 64)	61 (55, 66)	0.409[a]
**Preoperative PSA, ng/mL [median (Q1, Q3)]**	7.6 (5.3, 12)	8 (5.3, 12)	7.7 (5.4, 12)	7.3 (5.2, 12)	0.684[a]
**RP Gleason score, No. (%)**					
≤3 + 4	459 (53.8)	155 (55.0)	164 (56.6)	140 (49.8)	0.133[b]
4 + 3	171 (20.0)	56 (19.9)	46 (15.9)	69 (24.6)	
≥8	223 (26.1)	71 (25.2)	80 (27.6)	72 (25.6)	
**Extraprostatic extension, No. (%)**	359 (42.1)	92 (32.7)	122 (42.1)	145 (51.6)	<0.001[c]
**Seminal vesicle invasion, No. (%)**	238 (27.8)	62 (22.0)	81 (27.8)	95 (33.7)	0.008[c]
**Positive surgical margins, No. (%)**	499 (58.4)	153 (54.3)	178 (61.2)	168 (59.6)	0.216[c]
**Lymph node invasion, No. (%)**	49 (6.2)	9 (3.4)	14 (5.2)	26 (10.3)	0.005[c]
**Follow-up for DM status, years [median (Q1, Q3)]**	8.5 (5.4, 12)	8.5 (5.8, 12)	8.2 (5.1, 11)	8.8 (5.7, 12)	0.352[a]
**Follow-up for OS, years [median (Q1, Q3)]**	8.7 (5.7, 12)	8.8 (6, 12)	8.3 (5.3, 11)	9 (6, 12)	0.297[a]

[a]Kruskal-Wallis test; [b]Chi-squared test; [c]Fisher's exact test;

Supplementary Table 5: Patient characteristics table META855 cohort by CEG signature tertiles. Statistical tests used to obtain two-sided p-values (no multiple testing adjustment) are given below the table. Full data used to generate this table, and exact p-values provided in source data file for supplement.

Variables	Overall	CEG-tertile			P-value
		0%-33%	33%-67%	67%-100%	
**Total, No. (%)**	855 (100.0)	282 (33.0)	291 (34.0)	282 (33.0)	
**Race, No. (%)**					
White	731 (85.8)	235 (83.6)	255 (87.9)	241 (85.8)	0.679[b]
Black	104 (12.2)	39 (13.9)	30 (10.3)	35 (12.5)	
Other	17 (2.0)	7 (2.5)	5 (1.7)	5 (1.8)	
**Age, years [median (Q1, Q3)]**	60 (55, 65)	60 (54, 65)	61 (55, 64)	60 (55, 66)	0.713[a]
**Preoperative PSA, ng/mL [median (Q1, Q3)]**	7.6 (5.3, 12)	7.3 (5.1, 13)	7.7 (5.4, 11)	7.5 (5.3, 12)	0.880[a]
**RP Gleason score, No. (%)**					
≤3 + 4	459 (53.8)	150 (53.4)	165 (56.7)	144 (51.2)	0.606[b]
4 + 3	171 (20.0)	55 (19.6)	52 (17.9)	64 (22.8)	
≥8	223 (26.1)	76 (27.0)	74 (25.4)	73 (26.0)	
**Extraprostatic extension, No. (%)**	359 (42.1)	122 (43.4)	109 (37.6)	128 (45.6)	0.136[c]
**Seminal vesicle invasion, No. (%)**	238 (27.8)	81 (28.7)	72 (24.7)	85 (30.1)	0.328[c]
**Positive surgical margins, No. (%)**	499 (58.4)	162 (57.4)	173 (59.5)	164 (58.2)	0.895[c]
**Lymph node invasion, No. (%)**	49 (6.2)	13 (5.0)	18 (6.6)	18 (7.1)	0.586[c]
**Follow-up for DM status, years [median (Q1, Q3)]**	8.5 (5.4, 12)	8.2 (5.1, 11)	8.6 (5.4, 12)	8.8 (5.9, 12)	0.439[a]
**Follow-up for OS, years [median (Q1, Q3)]**	8.7 (5.7, 12)	8.4 (5.3, 12)	8.8 (5.7, 12)	8.9 (6, 12)	0.615[a]

[a]Kruskal-Wallis test; [b]Chi-squared test; [c]Fisher's exact test;

Supplementary Table 6: Univariable and multivariable analysis for biochemical recurrence using META855 cohort for APC signature tertiles. Two-sided p-values obtained using Gray's test with no multiple testing adjustment. Full data used to generate this table, and exact p-values provided in source data file for supplement.

Variables	Univariable		Multivariable	
	sHR (95% CI)	P-value	sHR (95% CI)	P-value
**APC-tertiles**				
0%-33%	ref	1	ref	1
33%-67%	1.39 (1.07 - 1.79)	0.01*	1.29 (0.96 - 1.73)	0.09
67%-100%	1.52 (1.19 - 1.95)	<0.001*	1.36 (1.00 - 1.83)	0.05*
**Log2(PSA)**	1.15 (1.03 - 1.29)	0.01*	1.15 (1.02 - 1.30)	0.02*
**RP Gleason score**				
≤3 + 4	ref	1	ref	1
4 + 3	1.34 (1.03 - 1.75)	0.03*	1.40 (1.05 - 1.88)	0.02*
≥8	1.92 (1.51 - 2.44)	<0.001*	1.99 (1.51 - 2.62)	<0.001*
**Positive surgical margins**	1.41 (1.13 - 1.76)	0.002*	1.59 (1.24 - 2.03)	<0.001*
**Extraprostatic extension**	1.37 (1.12 - 1.67)	0.002*	1.17 (0.91 - 1.51)	0.21
**Seminal vesicle invasion**	1.54 (1.24 - 1.92)	<0.001*	1.38 (1.06 - 1.78)	0.02*
**Lymph node invasion**	2.07 (1.39 - 3.09)	<0.001*	1.56 (0.99 - 2.47)	0.06

* p < 0.05.

sHR = Subdistribution hazard ratio; Multivariable Fine-Gray models were fitted.

Supplementary Table 7: Univariable and multivariable analysis for metastasis using META855 cohort for APC signature tertiles. Two-sided p-values obtained using Gray's test with no multiple testing adjustment. Full data used to generate this table, and exact p-values provided in source data file for supplement.

Variables	Univariable		Multivariable	
	sHR (95% CI)	P-value	sHR (95% CI)	P-value
**APC-tertiles**				
0%-33%	ref	1	ref	1
33%-67%	1.38 (0.76 - 2.49)	0.28	1.19 (0.63 - 2.25)	0.60
67%-100%	2.15 (1.25 - 3.71)	0.006*	1.51 (0.81 - 2.83)	0.20
**Log2(PSA)**	1.29 (1.02 - 1.63)	0.03*	1.05 (0.82 - 1.34)	0.70
**RP Gleason score**				
≤3 + 4	ref	1	ref	1
4 + 3	3.11 (1.66 - 5.80)	<0.001*	2.31 (1.18 - 4.50)	0.01*
≥8	6.07 (3.59 - 10.28)	<0.001*	4.03 (2.22 - 7.34)	<0.001*
**Positive surgical margins**	1.06 (0.68 - 1.64)	0.80	1.21 (0.73 - 1.99)	0.46
**Extraprostatic extension**	3.40 (2.13 - 5.42)	<0.001*	2.35 (1.29 - 4.27)	0.005*
**Seminal vesicle invasion**	2.74 (1.79 - 4.18)	<0.001*	1.68 (1.01 - 2.77)	0.04*
**Lymph node invasion**	5.91 (3.52 - 9.91)	<0.001*	3.03 (1.70 - 5.41)	<0.001*

* $p < 0.05$.

sHR = Subdistribution hazard ratio; Multivariable Fine-Gray models were fitted.

Supplementary Table 8: Univariable and multivariable analysis for biochemical recurrence using META855 cohort for CEG signature tertiles. Two-sided p-values obtained using Gray's test with no multiple testing adjustment. Full data used to generate this table, and exact p-values provided in source data file for supplement.

Variables	Univariable		Multivariable	
	sHR (95% CI)	P-value	sHR (95% CI)	P-value
**CEG_tertile**				
0%-33%	ref	1	ref	1
33%-67%	0.86 (0.67 - 1.11)	0.26	0.80 (0.60 - 1.08)	0.14
67%-100%	1.17 (0.92 - 1.48)	0.20	1.13 (0.86 - 1.49)	0.38
**Log2(PSA)**	1.15 (1.03 - 1.29)	0.01*	1.15 (1.02 - 1.30)	0.02*
**RP Gleason score**				
≤3 + 4	ref	1	ref	1
4 + 3	1.34 (1.03 - 1.75)	0.03*	1.39 (1.04 - 1.86)	0.03*
≥8	1.92 (1.51 - 2.44)	<0.001*	2.00 (1.51 - 2.63)	<0.001*
**Positive surgical margins**	1.41 (1.13 - 1.76)	0.002*	1.63 (1.28 - 2.09)	<0.001*
**Extraprostatic extension**	1.37 (1.12 - 1.67)	0.002*	1.21 (0.95 - 1.55)	0.13
**Seminal vesicle invasion**	1.54 (1.24 - 1.92)	<0.001*	1.40 (1.08 - 1.81)	0.01*
**Lymph node invasion**	2.07 (1.39 - 3.09)	<0.001*	1.65 (1.04 - 2.62)	0.03*

* $p < 0.05$.

sHR = Subdistribution hazard ratio; Multivariable Fine-Gray models were fitted.

Supplementary Table 9: Univariable and multivariable analysis for metastasis using META855 cohort for CEG signature tertiles. Two-sided p-values obtained using Gray's test with no multiple testing adjustment. Full data used to generate this table, and exact p-values provided in source data file for supplement.

Variables	Univariable		Multivariable	
	sHR (95% CI)	P-value	sHR (95% CI)	P-value
**CEG_tertile**				
0%-33%	ref	1	ref	1
33%-67%	0.84 (0.47 - 1.49)	0.56	0.66 (0.34 - 1.28)	0.22
67%-100%	1.51 (0.91 - 2.49)	0.11	1.43 (0.85 - 2.41)	0.18
**Log2(PSA)**	1.29 (1.02 - 1.63)	0.03*	1.03 (0.81 - 1.31)	0.79
**RP Gleason score**				
≤3 + 4	ref	1	ref	1
4 + 3	3.11 (1.66 - 5.80)	<0.001*	2.29 (1.18 - 4.47)	0.01*
≥8	6.07 (3.59 - 10.28)	<0.001*	4.09 (2.27 - 7.38)	<0.001*
**Positive surgical margins**	1.06 (0.68 - 1.64)	0.80	1.23 (0.77 - 1.99)	0.39
**Extraprostatic extension**	3.40 (2.13 - 5.42)	<0.001*	2.47 (1.39 - 4.38)	0.002*
**Seminal vesicle invasion**	2.74 (1.79 - 4.18)	<0.001*	1.65 (1.01 - 2.70)	0.05*
**Lymph node invasion**	5.91 (3.52 - 9.91)	<0.001*	3.31 (1.82 - 6.05)	<0.001*

* $p < 0.05$.

sHR = Subdistribution hazard ratio; Multivariable Fine-Gray models were fitted.
