# Supplementary material for: Spatial multi-omics identifies aggressive prostate cancer signatures highlighting pro-inflammatory chemokine activity in the tumor microenvironment
Source: Nat Commun. 2025 Nov 19;16:10160. doi: 10.1038/s41467-025-65161-9 (PMC12630738; doi:10.1038/s41467-025-65161-9)
Supplement: Supplementary file 3 — Reporting Summary [file 41467_2025_65161_MOESM3_ESM.pdf]

nature portfolio | reporting summary

nature portfolio

Corresponding author(s): Sebastian Krossa

Last updated by author(s): Jul 24, 2025

Reporting Summary

Nature Portfolio wishes to improve the reproducibility of the work that we publish. This form provides structure for consistency and transparency in reporting. For further information on Nature Portfolio policies, see our Editorial Policies and the Editorial Policy Checklist.

Statistics

For all statistical analyses, confirm that the following items are present in the figure legend, table legend, main text, or Methods section.

n/a	Confirmed	
☐	☒	The exact sample size (*n*) for each experimental group/condition, given as a discrete number and unit of measurement
☐	☒	A statement on whether measurements were taken from distinct samples or whether the same sample was measured repeatedly
☐	☒	The statistical test(s) used AND whether they are one- or two-sided<br>*Only common tests should be described solely by name; describe more complex techniques in the Methods section.*
☐	☒	A description of all covariates tested
☐	☒	A description of any assumptions or corrections, such as tests of normality and adjustment for multiple comparisons
☐	☒	A full description of the statistical parameters including central tendency (e.g. means) or other basic estimates (e.g. regression coefficient) AND variation (e.g. standard deviation) or associated estimates of uncertainty (e.g. confidence intervals)
☐	☒	For null hypothesis testing, the test statistic (e.g. *F*, *t*, *r*) with confidence intervals, effect sizes, degrees of freedom and *P* value noted<br>*Give P values as exact values whenever suitable.*
☒	☐	For Bayesian analysis, information on the choice of priors and Markov chain Monte Carlo settings
☒	☐	For hierarchical and complex designs, identification of the appropriate level for tests and full reporting of outcomes
☐	☒	Estimates of effect sizes (e.g. Cohen's *d*, Pearson's *r*), indicating how they were calculated

Our web collection on statistics for biologists contains articles on many of the points above.

Software and code

Policy information about availability of computer code

Data collection	RNAseq (ST & bulk): NextSeq Control Software (v4 Illumina); MSI: FlexImaging (Version 5.1.46.0_1455_51, Bruker Daltonics), FlexControl (Version 4.0.46.0_867_879, Bruker Daltonics), Microscopic images: Olympus VS200 ASW (3.3 Build 24382) and OLYMPUS VS-ASW (2.9 Build 13753), NMR data: TopSpin (3.5.6, Bruker BioSpin)
Data analysis	Spatial transcriptomics data were processed using 10x genomics space ranger software package (version 1.0.0) with human reference transcriptome GRCh38 version 3.0.0; Microscopic images were analysed and annotated with open source software QuPath (version >=0.2.3); Processing scripts available on github: https://github.com/sekro/qupath_scripts , https://github.com/sekro/spatial_transcriptomics_toolbox; Python 3.8.0 with packages: anndata (0.9.2), goatools (1.1.6), matplotlib (3.7.4), nmrglue (0.9), numpy (1.22.4), opencv-python (4.5.4.58), pandas (2.0.3), pyicoshift (0.0.1), scanpy (1.9.8), scipy (1.10.1), seaborn (0.13.2), scikit-learn (1.0.1), squidpy (1.2.3), stereoscope (0.2.0), tifffile (2022.10.10); RStudio (2024.12.1 Build 563) with R 4.4.2 and libraries: ggsurvfit (1.1.0), gtsummary (2.0.4), survival (3.7-0), survivalAnalysis (0.3.0), survminer (0.5.0); MSI data: SCiLS lab Pro (Version 2024a, Bruker Daltonics), MIIT (the Multi-omics Imaging Integration Toolset, https://github.com/mwess/miit, v0.0.3-rc1)

For manuscripts utilizing custom algorithms or software that are central to the research but not yet described in published literature, software must be made available to editors and reviewers. We strongly encourage code deposition in a community repository (e.g. GitHub). See the Nature Portfolio guidelines for submitting code & software for further information.

Data

Policy information about availability of data

All manuscripts must include a data availability statement. This statement should provide the following information, where applicable:

- Accession codes, unique identifiers, or web links for publicly available datasets
- A description of any restrictions on data availability
- For clinical datasets or third party data, please ensure that the statement adheres to our policy

Bulk and spatial transcriptomics data are available upon request through Federated European Genome Phenome Archive (FEGA) Norway data access committee with accession number EGAC50000000277 and bundled under study EGAS50000000413. Access will only be granted after the following steps have been achieved; 1. the data requester and the intended use of the data must comply with GDPR regulation, Norwegian law, and the specific patient consent, 2. data sharing with the specific data requester must be approved by the regional ethical committee (REC) in Norway, 3. the Data Protection Impact Assessment (DPIA) may require revision and 4. there must be a signed data transfer agreement between the institution of the data requester and NTNU. Depending on the intended use of the data, the data requester can also be required to establish a collaboration agreement with NTNU prior to data sharing.

Research involving human participants, their data, or biological material

Policy information about studies with human participants or human data. See also policy information about sex, gender (identity/presentation), and sexual orientation and race, ethnicity and racism.

Reporting on sex and gender	Not relevant for this study on prostate cancer as only biological males have a prostate.
Reporting on race, ethnicity, or other socially relevant groupings	We had no access to ethnicity or any lifestyle data except the patient's hospital record (Biobank1, St Olav University Hospital, Trondheim, Norway) associated with the clinical follow-up data of potential treatment after surgery and mortality. The patients used in this cohort had no records of additional genetic background.
Population characteristics	see above, clinical parameters are reported in Supplementary Table 1
Recruitment	The study utilized human prostate tissue samples obtained from PCa patients who gave informed written consent before undergoing radical prostatectomy at St. Olav's Hospital in Trondheim between 2008 and 2016. The tissue from patients included in this study were retrieved from our local hospital biobank (Biobank1, St Olav University Hospital, Trondheim, Norway) established in 2008. Patients were chosen based on various clinical factors; large enough cancer (to extract two 3 mm cores) found within the 2 mm thick whole prostate tissue slice, recurrence status, no additional adjuvant treatment and a patient with longest follow-up time possible.
Ethics oversight	This research received approval from the regional ethical committee of Central Norway (identifier 2017/576) and adhered to both national and EU ethical regulations

Note that full information on the approval of the study protocol must also be provided in the manuscript.

Field-specific reporting

Please select the one below that is the best fit for your research. If you are not sure, read the appropriate sections before making your selection.

☒ Life sciences ☐ Behavioural & social sciences ☐ Ecological, evolutionary & environmental sciences

For a reference copy of the document with all sections, see nature.com/documents/nr-reporting-summary-flat.pdf

Life sciences study design

All studies must disclose on these points even when the disclosure is negative.

Sample size	Choosen sample sizes were the result of balancing sufficient n to allow robust analysis against costs and feasibility. Spatial -omics data is typically associated with substantially higher costs resulting in lower number of samples used as compared to bulk -omics data. We have ensured that sample sizes are comparable or higher than comparable studies. Further, each spatial data obtained from one sample contains multiple partially independent data points resulting in larger sample sizes depending on the grouping of these data points. We provide detailed sample sizes below. To validate our results in a larger patient cohort we have validated our finds using publicly available data from in total 1588 samples/patients.<br><br>Sample sizes of data obtained for this study:<br>Spatial data (Spatial transcriptomics, MSI, IHC): 8 patients (non-aggressive n=3, aggressive n=5 disease), 32 samples (4 per patient: non-aggressive n=12, aggressive n=20) resulting after data processing in:<br>spatial transcriptomics data points/spots: 19854 in total, on average 2482 (min 2112, max 2726) data points per patient, on average 620 (min 450, max 751) data points per sample<br>MSI: 19782 in total, on average 2473 (min 2083, max 2725) data points per patient, on average 618 (min 450, max 748) data points per sample<br>IHC: 746976 (LPS) and 718901 (LTA) cells in total, on average LPS: 93372 (min 75565, max 112515), LTA: 89862 (min 49174, max 120322) cells per patient, on average LPS: 23343 (min 13531, max 36188), LTA: 23963 (min 534, max 34661) cells per sample

	Bulk data (RNA-seq, NMR): 37 patients (non-aggressive n=10, aggressive n=27 disease), 174 samples (non-aggressive n=48, aggressive n=126 disease)<br><br>Samples sizes of public data used for validation:<br>GSE116918 cohort 248 cases (56 with biochemical recurrence)<br>TCGA-PRAD cohort 485 cases (99 with biochemical recurrence)<br>META855 cohort 855 cases (373 with biochemical recurrence, 85 with metastasis)
Data exclusions	Spatial transcriptomics data: Spots were excluded if they contained less than 50% tissue, more than 50% folded or low staining quality tissue, or more than 80% luminal space. Spots with less than 100 normalized total counts or less than 40 normalized number of detected genes per spot were excluded. Genes were only included if total raw count per gene over all spots was not zero and at least 10 counts for at least 10 spots were observed.<br>MSI data: MALDI-MSI raw data were binned/down sampled to 80% of its original datapoints in FlexImaging (Version 5.0, Bruker Daltonics). After co-registration to spatial transcriptomics data using MIIT spots that did not overlap with spatial trabscriptomics data were excluded.<br>IHC: 2 LTA-stained sections had to be excluded due partial sample loss during mounting.<br><br>Public data:<br>TCGA-PRAD: Only samples with RNA sequencing data and database fields 'Tissue Type' set to 'Tumor' and 'Preservation Mode' not set to 'FFPE' (GDC Data Release v42.0) were included.
Replication	No technical replication was performed in this study. Cancer tissue samples and sections are heterogeneous and serial sections are therefore never identical. True technical replication of experiments based on such samples are therefore not possible.<br>For each statistical test the input sample was either a spatial data point or whole tissue sample. Samples within the same group being tested against another group are considered as biological replicates to each other.
Randomization	This is a retrospective study where the clinical end-points were already known, and randomization of participants are therefore not relevant. The order of data collection was randomize for all methods to limit potential technical batch effects.
Blinding	Since this is a retrospective study, blinding was not necessary

Reporting for specific materials, systems and methods

We require information from authors about some types of materials, experimental systems and methods used in many studies. Here, indicate whether each material, system or method listed is relevant to your study. If you are not sure if a list item applies to your research, read the appropriate section before selecting a response.

Materials & experimental systems

n/a	Involved in the study
☐	☒ Antibodies
☒	☐ Eukaryotic cell lines
☒	☐ Palaeontology and archaeology
☒	☐ Animals and other organisms
☐	☒ Clinical data
☒	☐ Dual use research of concern
☒	☐ Plants

Methods

n/a	Involved in the study
☒	☐ ChIP-seq
☒	☐ Flow cytometry
☒	☐ MRI-based neuroimaging

Antibodies

Antibodies used	Anti-lipopolysaccharides (LPS) Abcam ab35654, clone 2D7/1, Lot No: GR3410325, anti-lipoteichoic acid (LTA) Thermo/Invitrogen MA1-7402, clone G43J, Agilent EnVision anti-mouse-HRP/DAB+ system
Validation	Anti-LPS and anti-LTA antibody dilutions were tested and validated against against fixed and embedded E. coli and B. subtilis cultures. anti-mouse-HRP was used according to manufacture's recommendations.

Clinical data

Policy information about clinical studies

All manuscripts should comply with the ICMJE guidelines for publication of clinical research and a completed CONSORT checklist must be included with all submissions.

Clinical trial registration	This was not a clinical trial study
Study protocol	This was not a clinical trial study
Data collection	The study utilized human prostate tissue samples obtained from PCa patients who gave informed written consent before undergoing radical prostatectomy at St. Olav's Hospital in Trondheim between 2008 and 2016. The tissue from patients included in this study

were retrieved from our local hospital biobank (Biobank1, St Olav University Hospital, Trondheim, Norway) established in 2008. Clinical data was obtained from the medical journal of each patient.

Outcomes

This is a retrospective study and none of the outcomes were therefore defined ahead. Based on disease progression during follow-up patients (n=10) that remained relapse-free for >10 years following surgery were grouped as non-aggressive PCa, while patients that either relapsed (n=16, biochemical recurrence, PSA > 0.2 ng/ml) or were persistent (n=11, PSA > 0.1 ng/ml) were grouped as aggressive PCa.

Plants

Seed stocks

n/a

Novel plant genotypes

n/a

Authentication

n/a
