# Supplementary material for: Spatial multi-omics identifies aggressive prostate cancer signatures highlighting pro-inflammatory chemokine activity in the tumor microenvironment
Source: Nat Commun. 2025 Nov 19;16:10160. doi: 10.1038/s41467-025-65161-9 (PMC12630738; doi:10.1038/s41467-025-65161-9)
Supplement: Supplementary file 4 — Description of Additional Supplementary Files [file 41467_2025_65161_MOESM4_ESM.pdf]

Description of Additional Supplementary Files

Supplementary Data 1(referenced in results):

Full results from gene ontology enrichment analysis performed during signature development.

Supplementary Data 2 (referenced in Figure 7 legend and results):

Full results from Pearson correlation between gene sets across 12 public data sets including correlations of individual public data sets.

Supplementary Data 3 (referenced in methods):

Sampling sites of all cores used for spatial analysis overlayed onto fresh/fresh-frozen (left column), and HES stained whole mount section 1 (center) and 2 (right, if available) images with identified cancer regions as indicated in respective legends. Cores are color-coded according to final core type after adjustment based on individual, per-core histopathology evaluation. Core IDs are given on fresh /fresh-frozen slide images.

Supplementary Data 4 (referenced in methods):

Table of identified mass spectrometry peaks with m/z, interval width and metabolite Name.

Supplementary Data 5 (referenced in methods):

Ion images of metabolites detected in negative mode using MALDI-TOF MSI (pages 1-5). Metabolites and m/z are annotated on each image. Shown are the root mean square (RMS) normalized highest intensity values in the +/- 400 ppm range around these m/z values. For each sample the total regions imaged at 30 µm x 30 µm were larger than the actual tissue (imaged region and tissue border indicated by outer and inner colored line, respectively) to capture the extent of potential delocalization of the respective molecule. The regions labeled M1-M8 were collected in the center of each slide (8 in total with 4 tissue samples each) to control for background signals.

Ion images of metabolites registered to Spatial transcriptomics (ST) spots using our Multi-Omics Imaging Integration Toolset (MIIT, pages 6-18). Spots are colored using the virids color map representing intensities from 0 to 0.67 covering the 0 to 0.99 quantile of the data as indicated by the color bar. Values above this are all represented by the color of the 0.99 quantile value 0.67.

(page 19) Mean root mean square normalized spectra of all MSI samples grouped by relapse status and off-tissue background spectra collected on each slide. Shown are the regions of the used metabolites in individual sub-plots. The +/- 400 ppm range used to determine the intensity for the ion images is indicated by the dashed vertical lines. To adjust for the pronounced background signals in the off-tissue regions, its mean spectrum was additionally normalized to the mean NEDC (221.1 m/z) on-tissue intensity. The peak-split observed for the mean spectra of ATP were due to a slight mass shift in one of the imaging batches.
