# Supplementary material for: Spatial multi-omics identifies aggressive prostate cancer signatures highlighting pro-inflammatory chemokine activity in the tumor microenvironment
Source: Nat Commun. 2025 Nov 19;16:10160. doi: 10.1038/s41467-025-65161-9 (PMC12630738; doi:10.1038/s41467-025-65161-9)
Supplement: Supplementary file 7 — Supplementary Data 3 [file 41467_2025_65161_MOESM7_ESM.pdf]

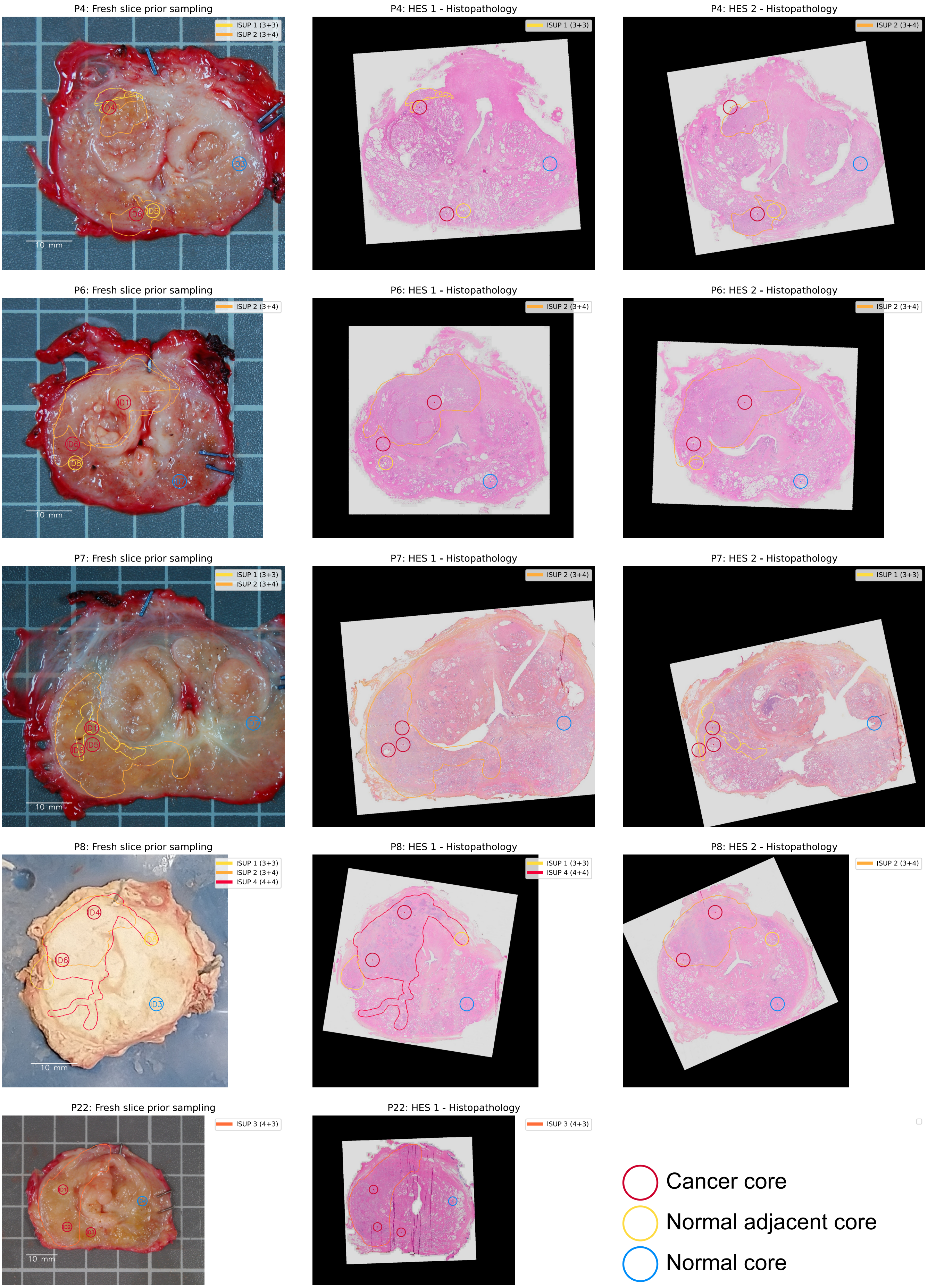

Sampling sites of all cores overlayed onto fresh/fresh-frozen (left column), and HES stained whole mount section 1 (center) and 2 (right, if availabale) images with identified cancer regions as indicated in respective legends. Cores are color-coded according to final core type after adjustment based on individual, per-core histopathology evaluation. Core IDs are given on fresh /fresh-frozen slide images.

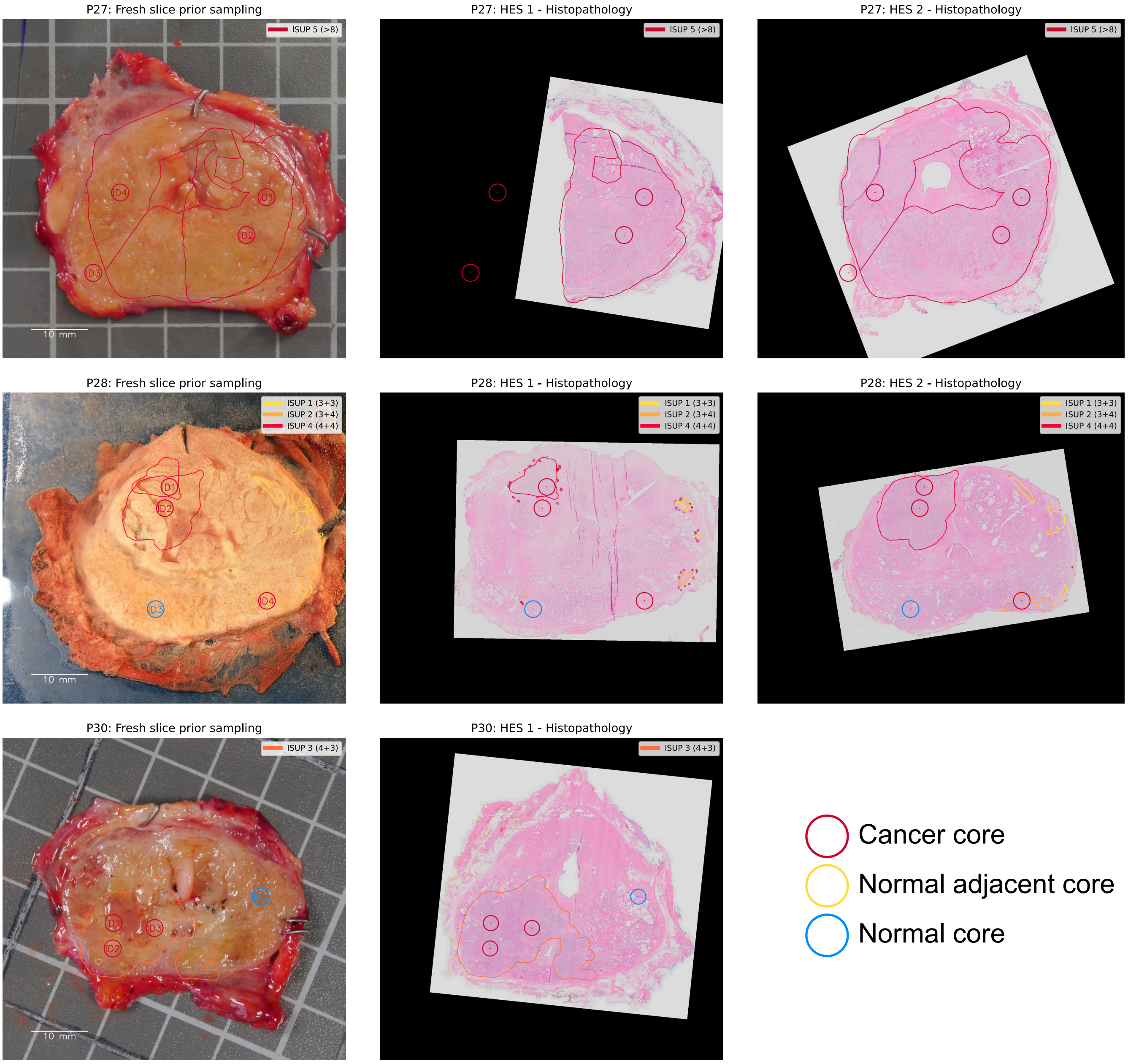

Sampling sites of all cores overlayed onto fresh/fresh-frozen (left column), and HES stained whole mount section 1 (center) and 2 (right, if availabale) images with identified cancer regions as indicated in respective legends. Cores are color-coded according to final core type after adjustment based on individual, per-core histopathology evaluation. Core IDs are given on fresh /fresh-frozen slide images.
