# Supplementary material for: Spatial multi-omics identifies aggressive prostate cancer signatures highlighting pro-inflammatory chemokine activity in the tumor microenvironment
Source: Nat Commun. 2025 Nov 19;16:10160. doi: 10.1038/s41467-025-65161-9 (PMC12630738; doi:10.1038/s41467-025-65161-9)
Supplement: Supplementary file 9 — Supplementary Data 5 [file 41467_2025_65161_MOESM9_ESM.pdf]

Taurine

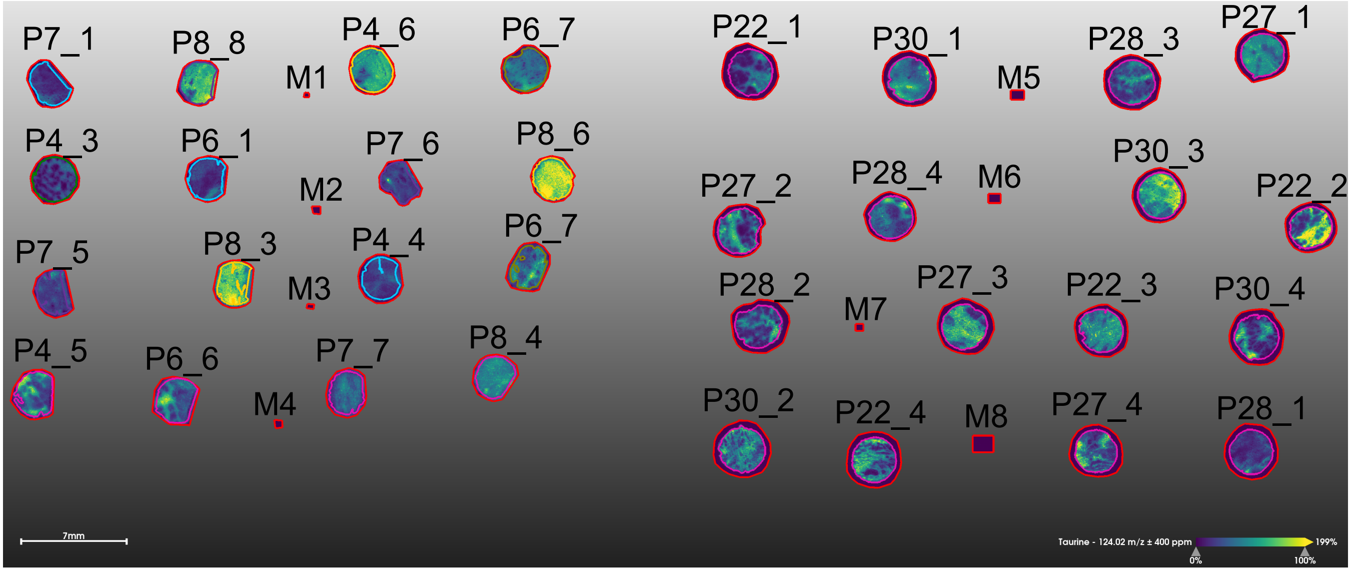

Aspartate

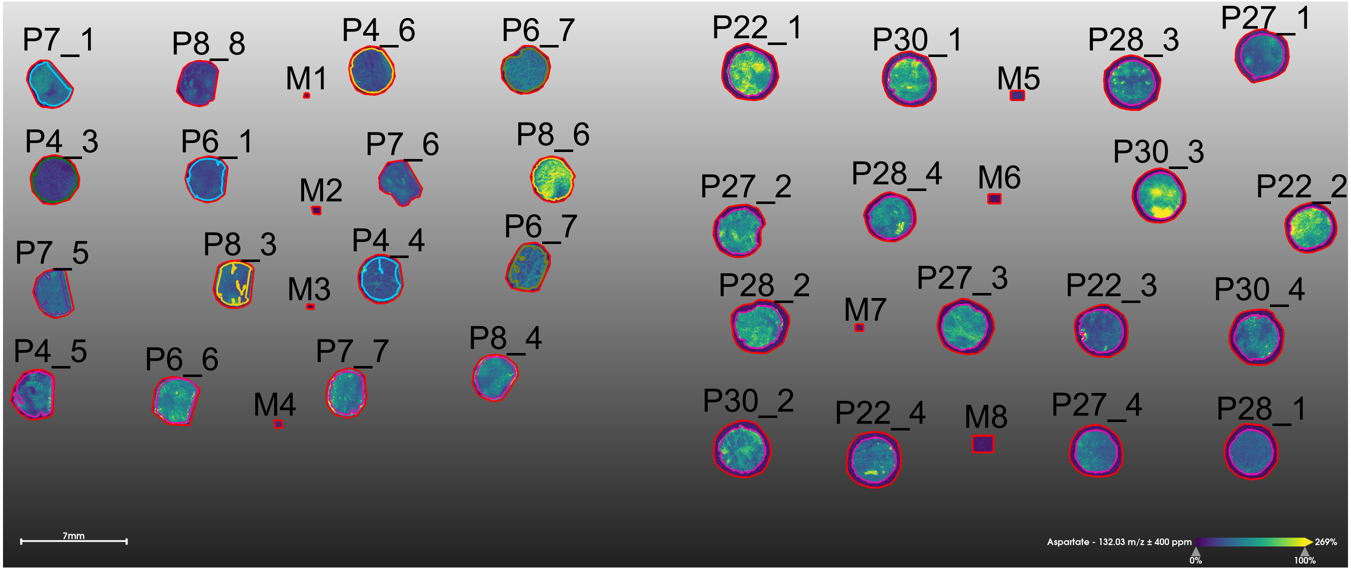

Ion images of metabolites detected in negative mode using MALDI-TOF MSI. Metabolites and m/z are annotated on each image. Shown are the root mean square (RMS) normalized highest intensity values in the +/- 400 ppm range around these m/z values. For each sample the total regions imaged at 30 µm x 30 µm were larger than the actual tissue (imaged region and tissue border indicated by outer and inner colored line, respectively) to capture the extent of potential delocalization of the respective molecule. The regions labeled M1-M8 were collected in the center of each slide (8 in total with 4 tissue samples each) to control for background signals.

Glutamine

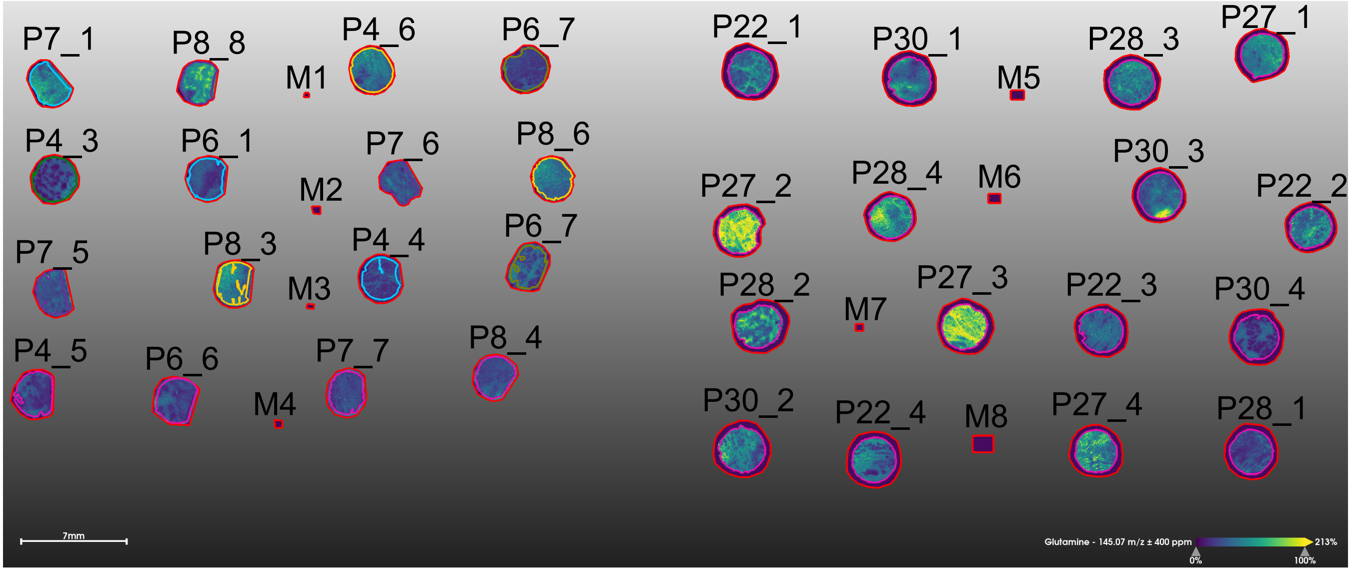

Glutamate

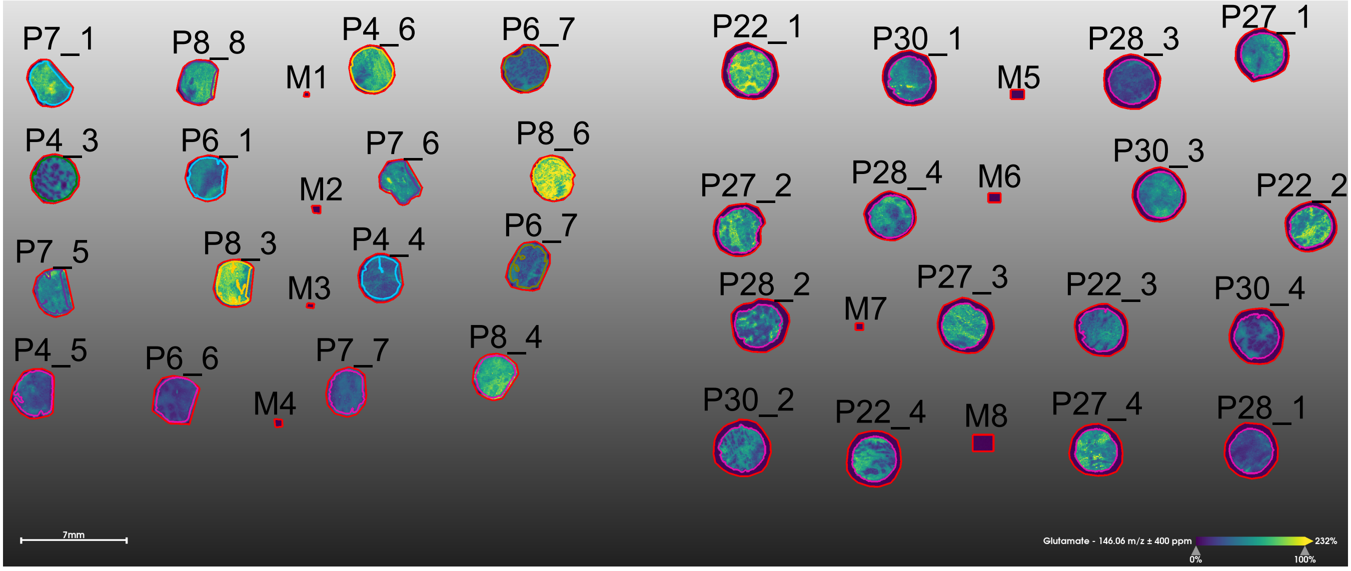

Urate

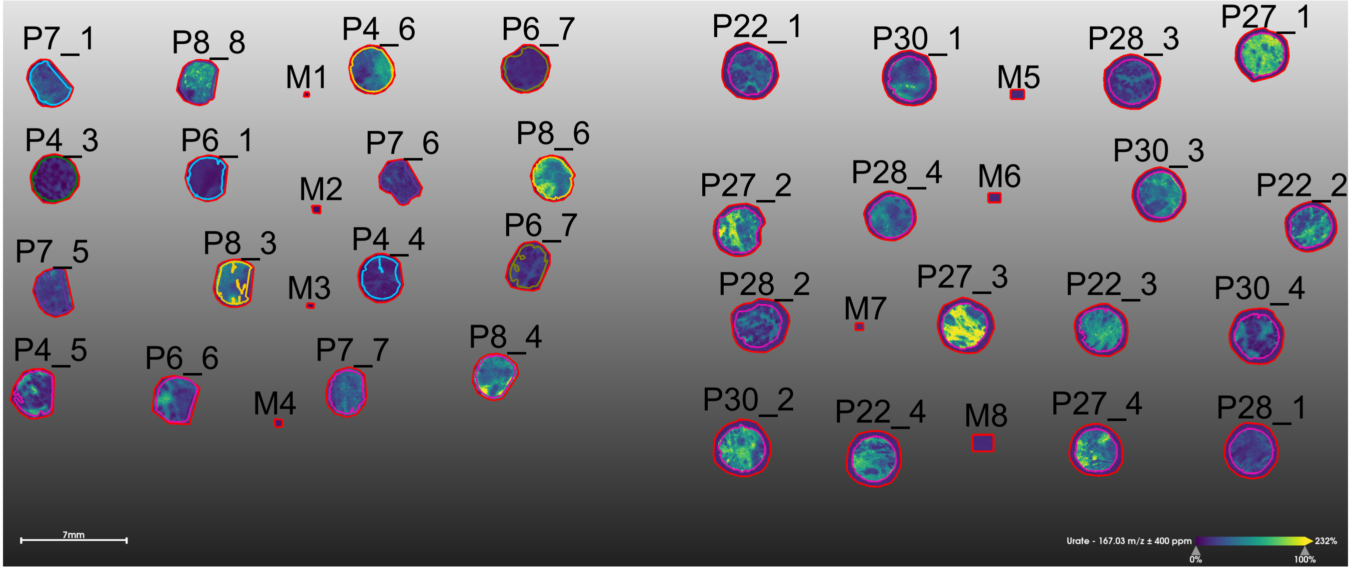

Ion images of metabolites detected in negative mode using MALDI-TOF MSI. Metabolites and m/z are annotated on each image. Shown are the root mean square (RMS) normalized highest intensity values in the +/- 400 ppm range around these m/z values. For each sample the total regions imaged at 30 µm x 30 µm were larger than the actual tissue (imaged region and tissue border indicated by outer and inner colored line, respectively) to capture the extent of potential delocalization of the respective molecule. The regions labeled M1-M8 were collected in the center of each slide (8 in total with 4 tissue samples each) to control for background signals.

NAA

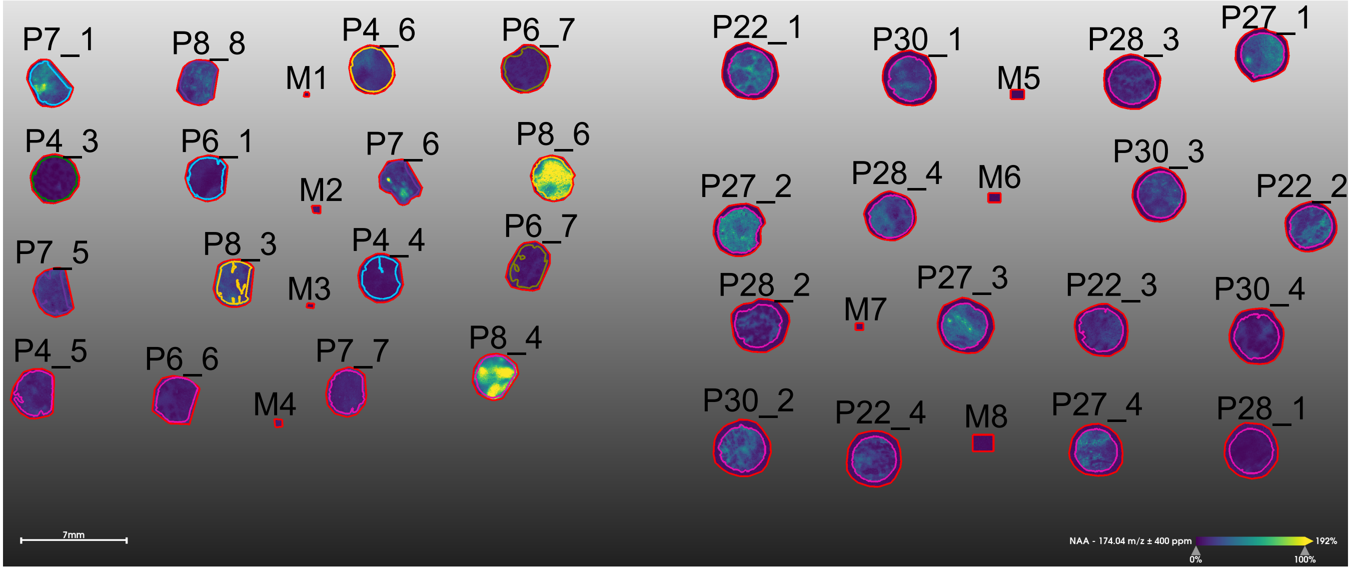

Zinc

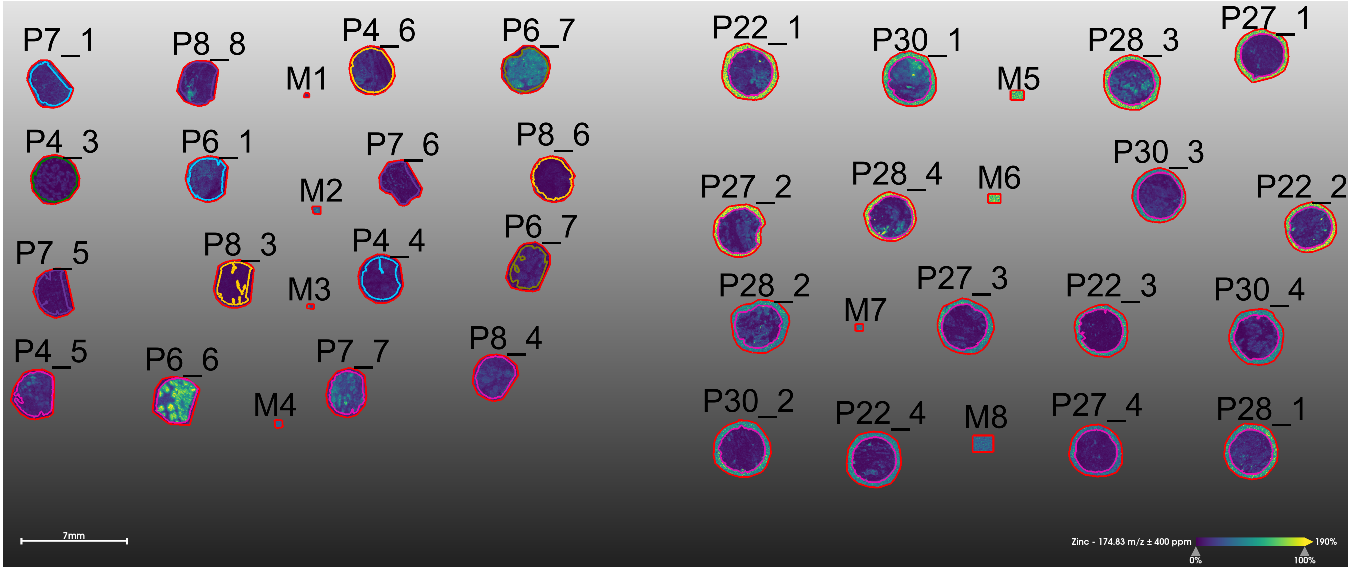

Citrate

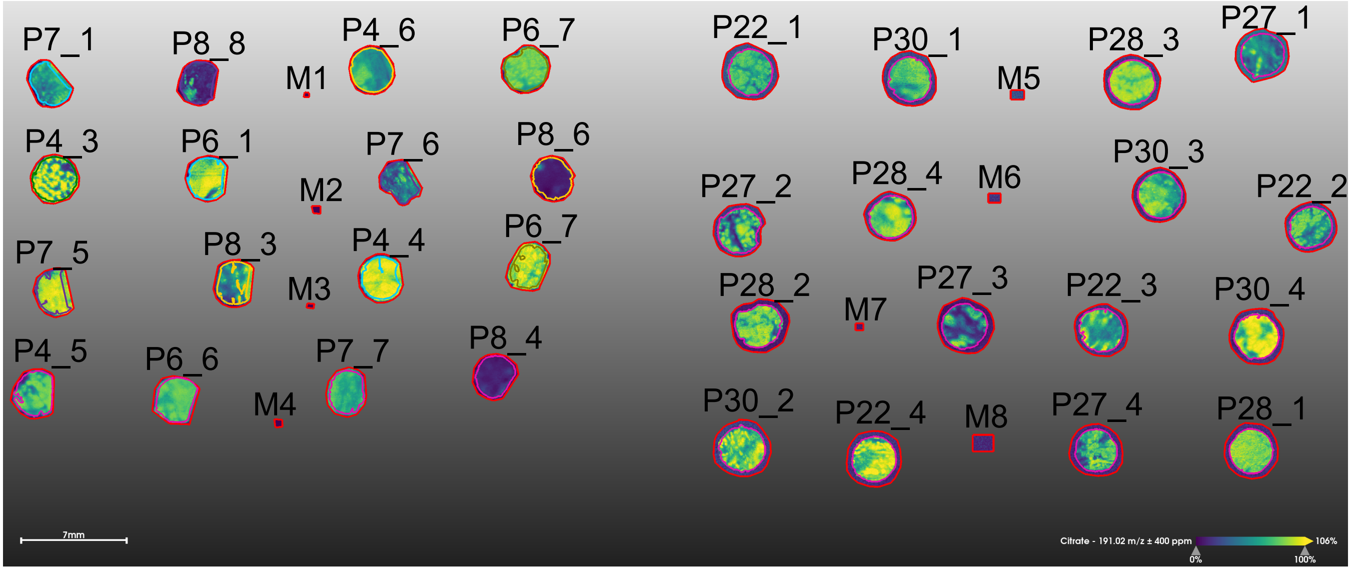

Ion images of metabolites detected in negative mode using MALDI-TOF MSI. Metabolites and m/z are annotated on each image. Shown are the root mean square (RMS) normalized highest intensity values in the +/- 400 ppm range around these m/z values. For each sample the total regions imaged at 30 µm x 30 µm were larger than the actual tissue (imaged region and tissue border indicated by outer and inner colored line, respectively) to capture the extent of potential delocalization of the respective molecule. The regions labeled M1-M8 were collected in the center of each slide (8 in total with 4 tissue samples each) to control for background signals.

Glucose

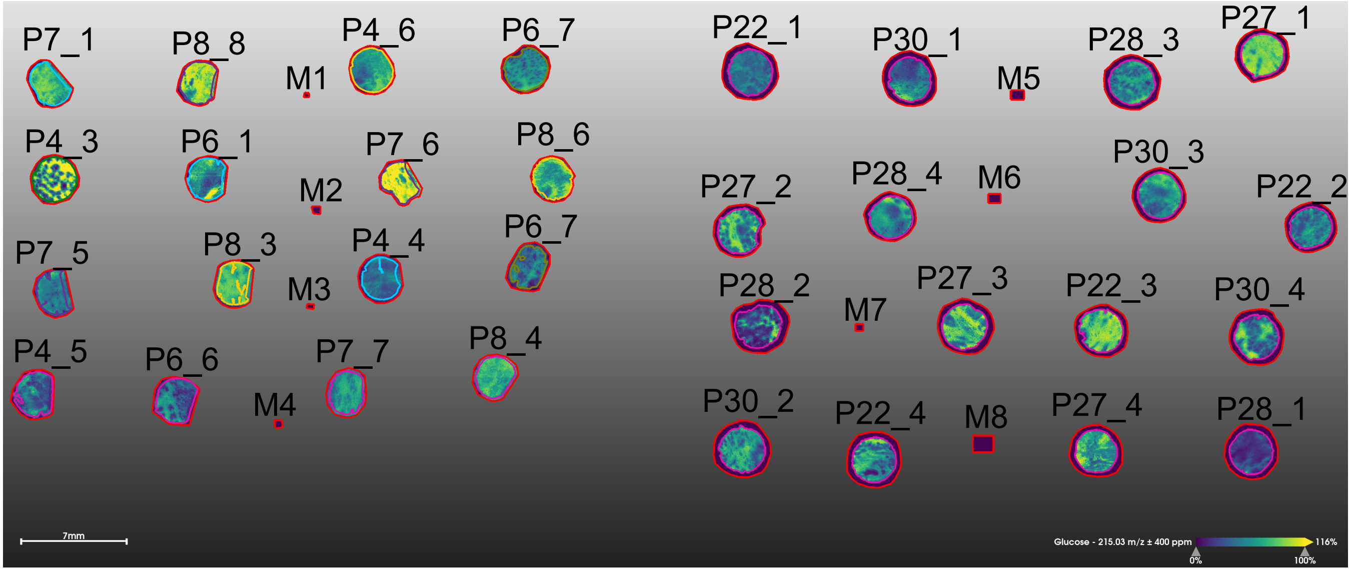

GSH

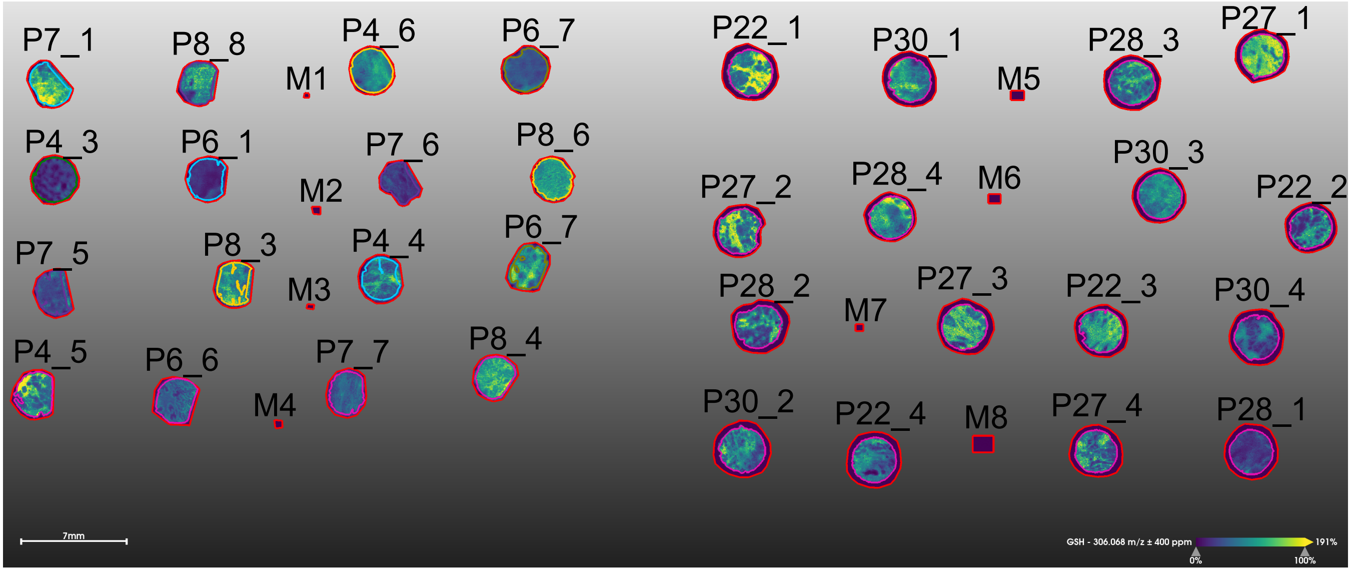

Ion images of metabolites detected in negative mode using MALDI-TOF MSI. Metabolites and m/z are annotated on each image. Shown are the root mean square (RMS) normalized highest intensity values in the +/- 400 ppm range around these m/z values. For each sample the total regions imaged at 30 µm x 30 µm were larger than the actual tissue (imaged region and tissue border indicated by outer and inner colored line, respectively) to capture the extent of potential delocalization of the respective molecule. The regions labeled M1-M8 were collected in the center of each slide (8 in total with 4 tissue samples each) to control for background signals.

AMP

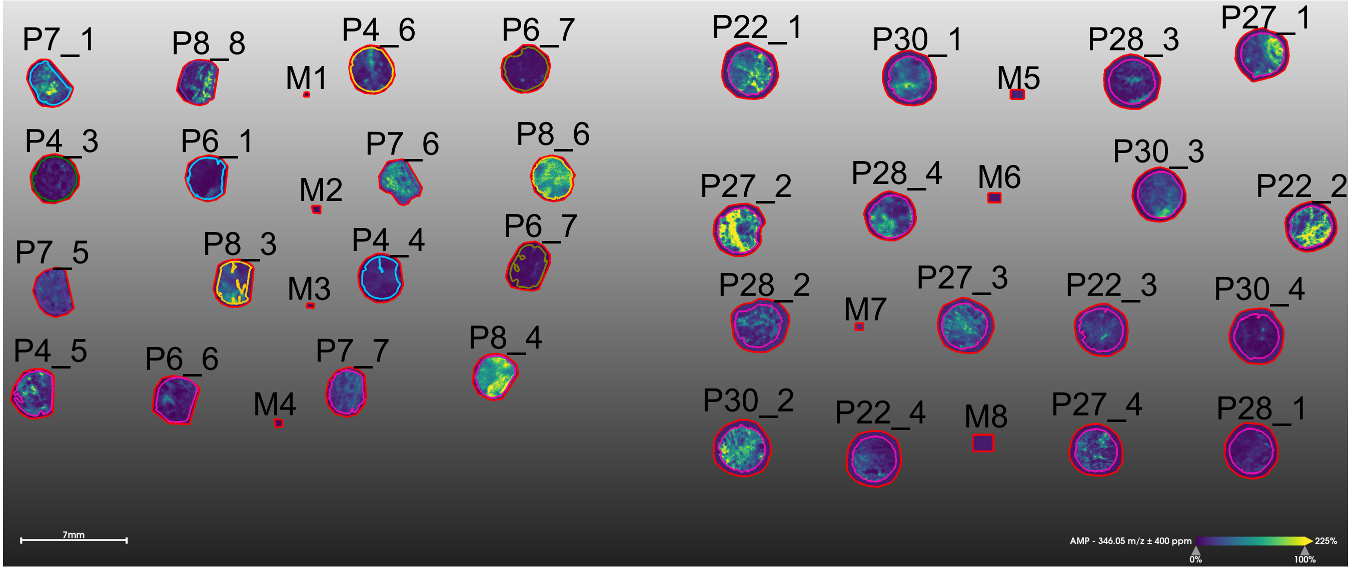

ADP

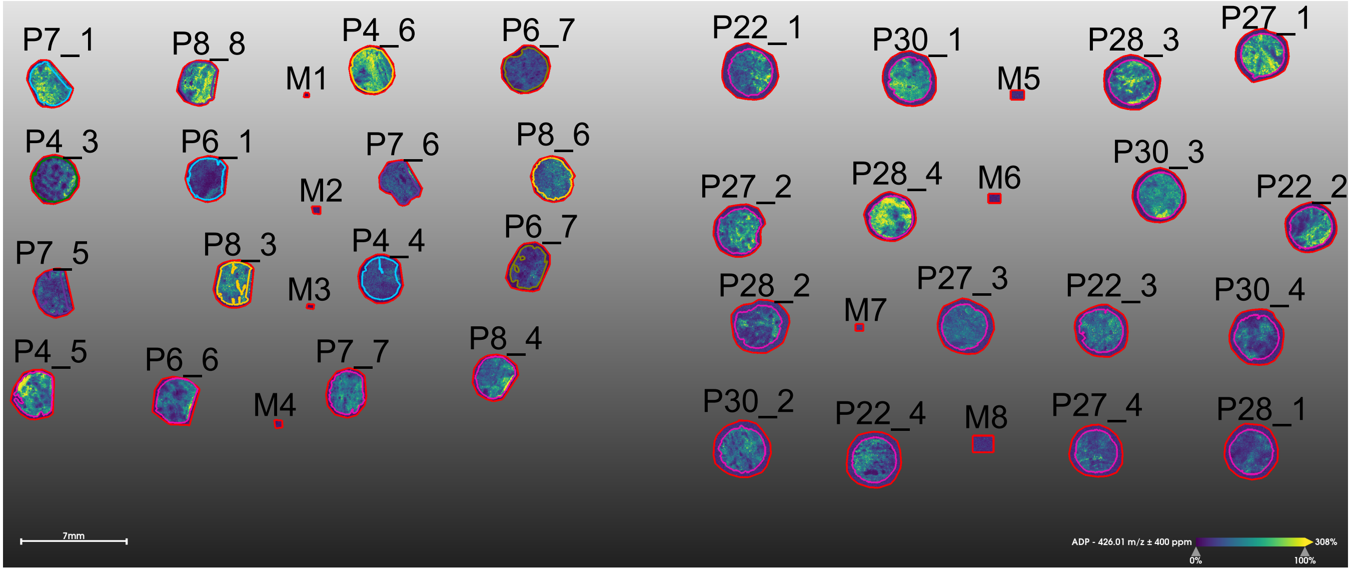

ATP

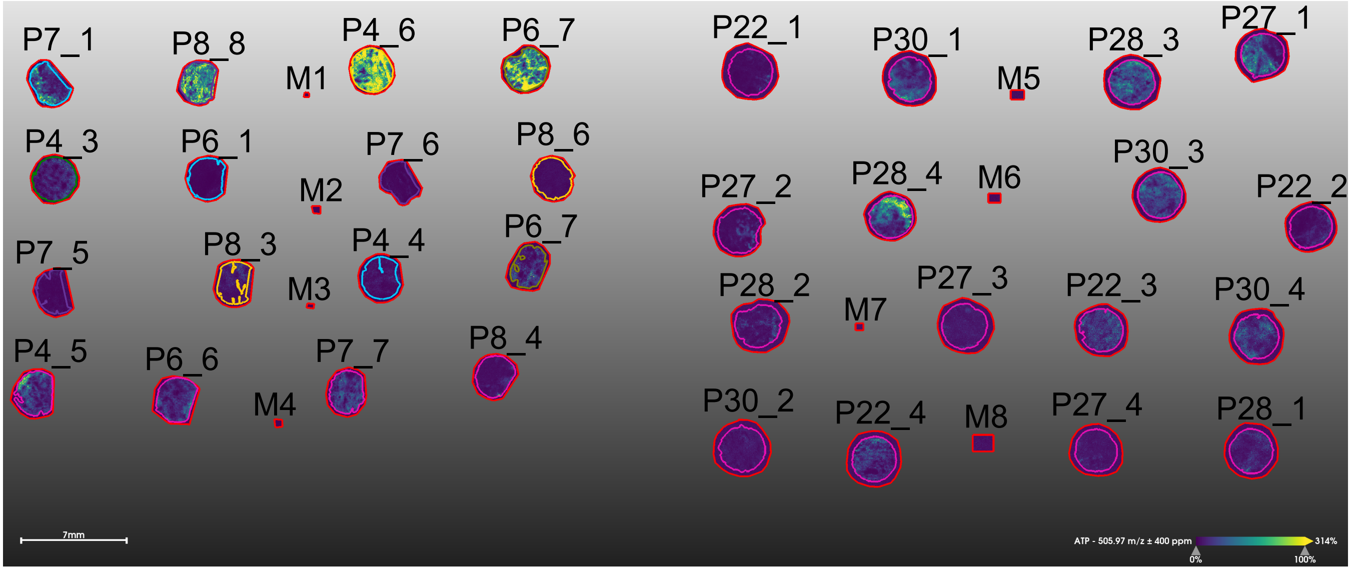

Ion images of metabolites detected in negative mode using MALDI-TOF MSI. Metabolites and m/z are annotated on each image. Shown are the root mean square (RMS) normalized highest intensity values in the +/- 400 ppm range around these m/z values. For each sample the total regions imaged at 30 µm x 30 µm were larger than the actual tissue (imaged region and tissue border indicated by outer and inner colored line, respectively) to capture the extent of potential delocalization of the respective molecule. The regions labeled M1-M8 were collected in the center of each slide (8 in total with 4 tissue samples each) to control for background signals.

Multi-Omics Imaging Integration Toolset (MIIT) registered MSI data of Taurine

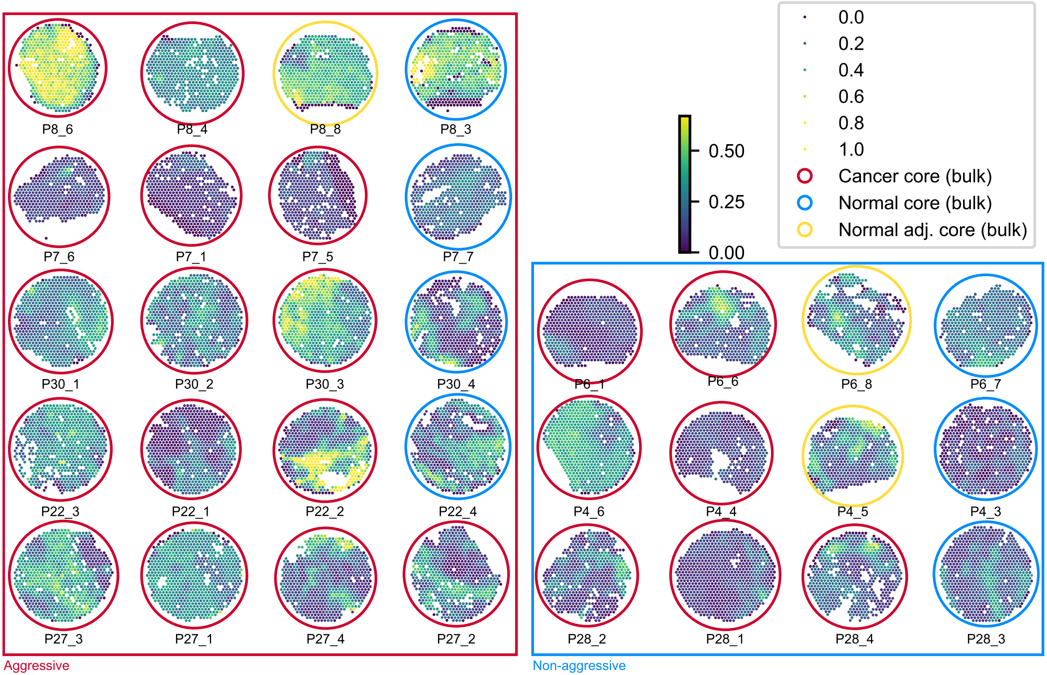

Ion images of Taurine (m/z 124.02 +/- 124.02 Da/ +/- 400 ppm, root mean square (RMS) normalized highest intensity) detected in negative mode using MALDI-TOF MSI were registered to Spatial transcriptomics (ST) spots using our Multi-Omics Imaging Integration Toolset (MIIT). Spots are colored using the virids color map representing intensities from 0 to 0.67 covering the 0 to 0.99 quantile of the data as indicated by the color bar. Values above this are all respresented by the color of the 0.99 quantile value 0.67.

Multi-Omics Imaging Integration Toolset (MIIT) registered MSI data of Aspartate

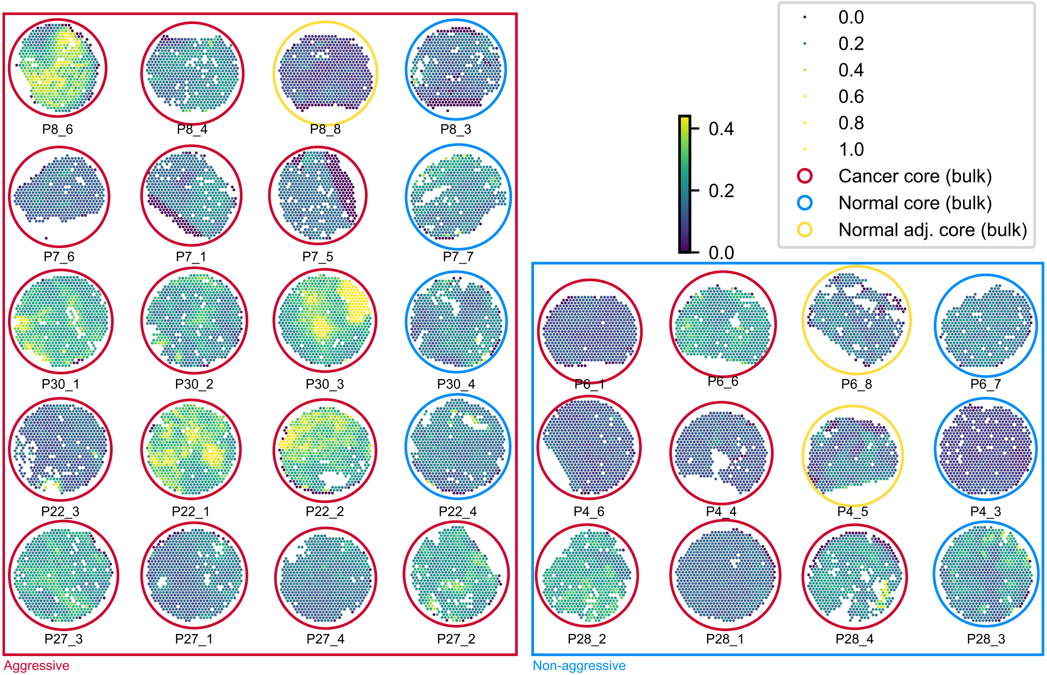

Ion images of Aspartate (m/z 132.03 +/- 132.03 Da/ +/- 400 ppm, root mean square (RMS) normalized highest intensity) detected in negative mode using MALDI-TOF MSI were registered to Spatial transcriptomics (ST) spots using our Multi-Omics Imaging Integration Toolset (MIIT). Spots are colored using the virids color map representing intensities from 0 to 0.44 covering the 0 to 0.99 quantile of the data as indicated by the color bar. Values above this are all respresented by the color of the 0.99 quantile value 0.44.

Multi-Omics Imaging Integration Toolset (MIIT) registered MSI data of Glutamine

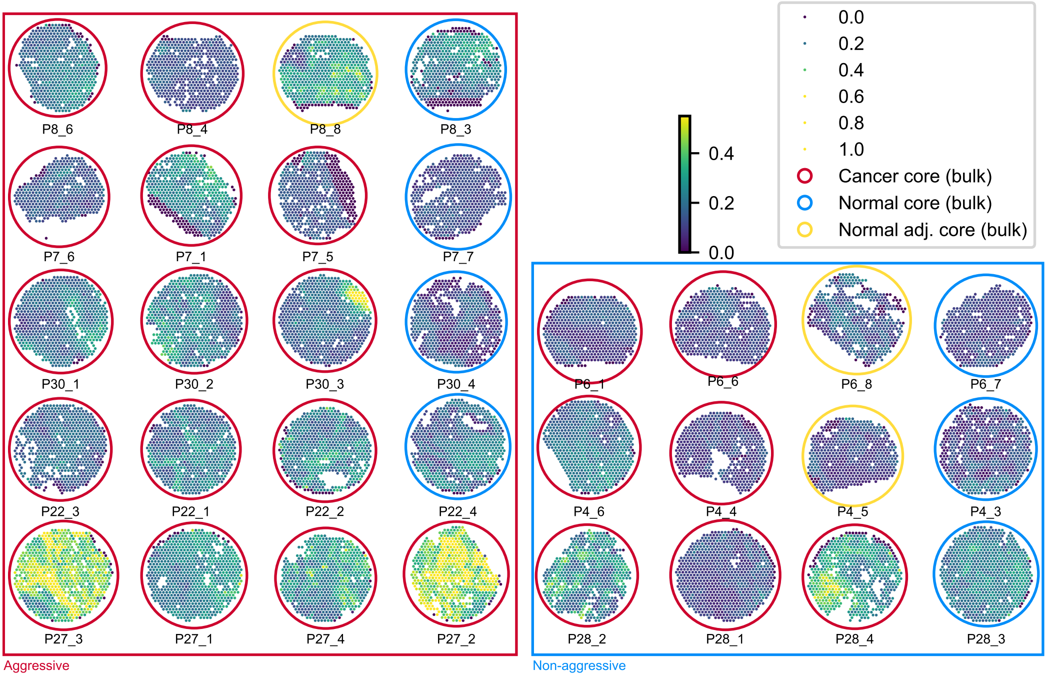

Ion images of Glutamine (m/z 145.07 +/- 145.07 Da/ +/- 400 ppm, root mean square (RMS) normalized highest intensity) detected in negative mode using MALDI-TOF MSI were registered to Spatial transcriptomics (ST) spots using our Multi-Omics Imaging Integration Toolset (MIIT). Spots are colored using the virids color map representing intensities from 0 to 0.55 covering the 0 to 0.99 quantile of the data as indicated by the color bar. Values above this are all respresented by the color of the 0.99 quantile value 0.55.

Multi-Omics Imaging Integration Toolset (MIIT) registered MSI data of Glutamate

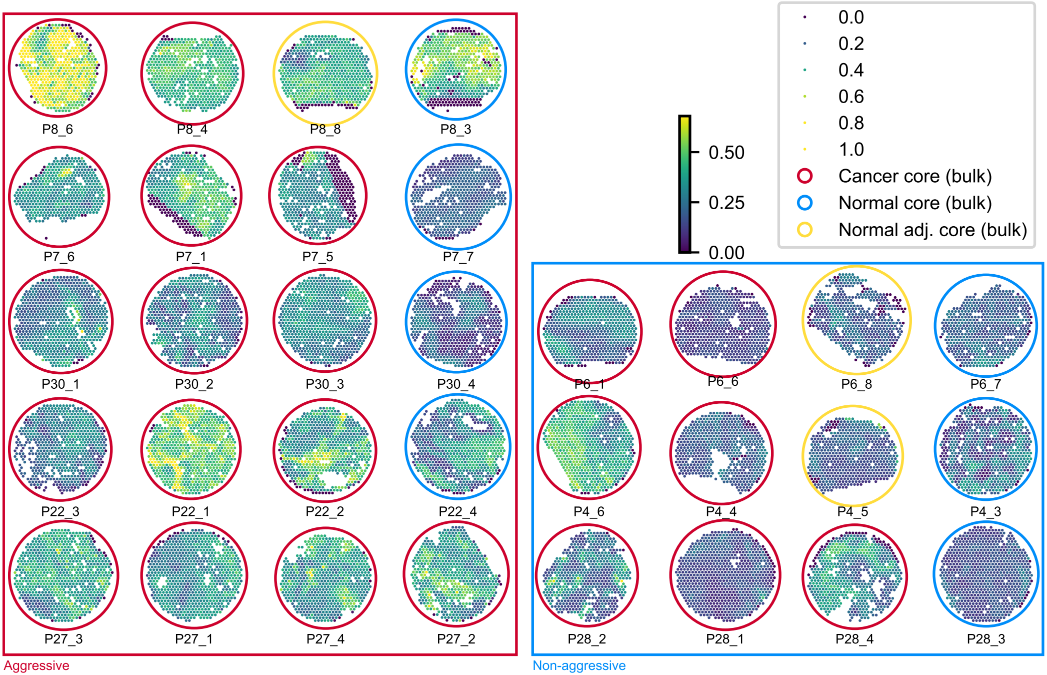

Ion images of Glutamate (m/z 146.06 +/- 146.06 Da/ +/- 400 ppm, root mean square (RMS) normalized highest intensity) detected in negative mode using MALDI-TOF MSI were registered to Spatial transcriptomics (ST) spots using our Multi-Omics Imaging Integration Toolset (MIIT). Spots are colored using the virids color map representing intensities from 0 to 0.68 covering the 0 to 0.99 quantile of the data as indicated by the color bar. Values above this are all respresented by the color of the 0.99 quantile value 0.68.

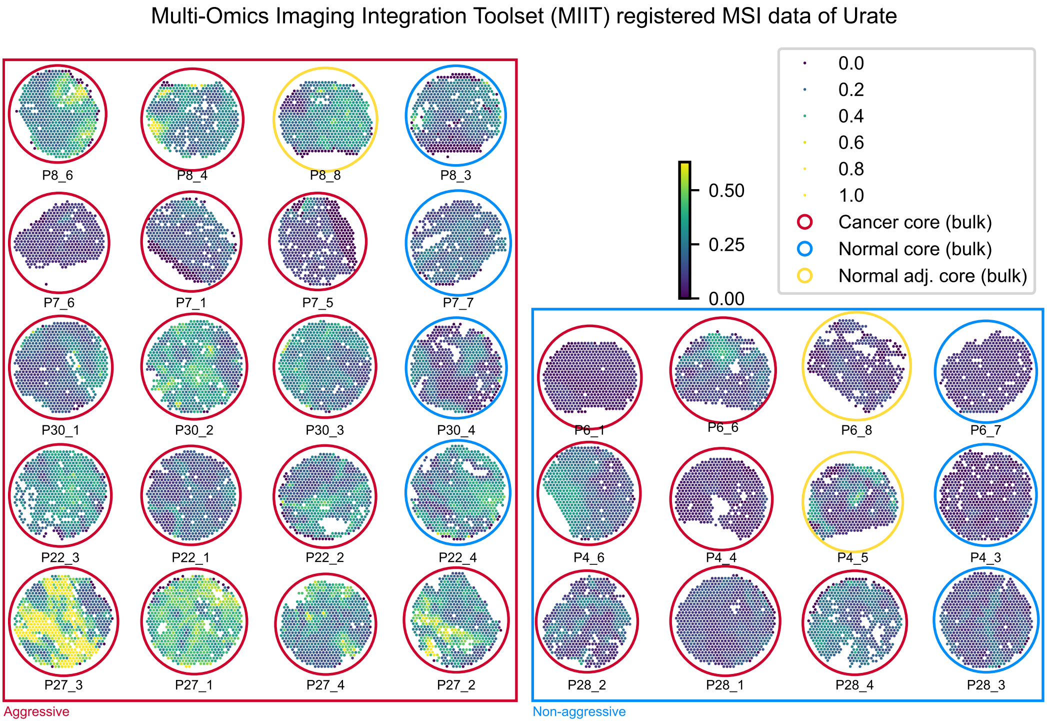

Ion images of Urate (m/z 167.03 +/- 167.03 Da/ +/- 400 ppm, root mean square (RMS) normalized highest intensity) detected in negative mode using MALDI-TOF MSI were registered to Spatial transcriptomics (ST) spots using our Multi-Omics Imaging Integration Toolset (MIIT). Spots are colored using the virids color map representing intensities from 0 to 0.63 covering the 0 to 0.99 quantile of the data as indicated by the color bar. Values above this are all respresented by the color of the 0.99 quantile value 0.63.

Multi-Omics Imaging Integration Toolset (MIIT) registered MSI data of NAA

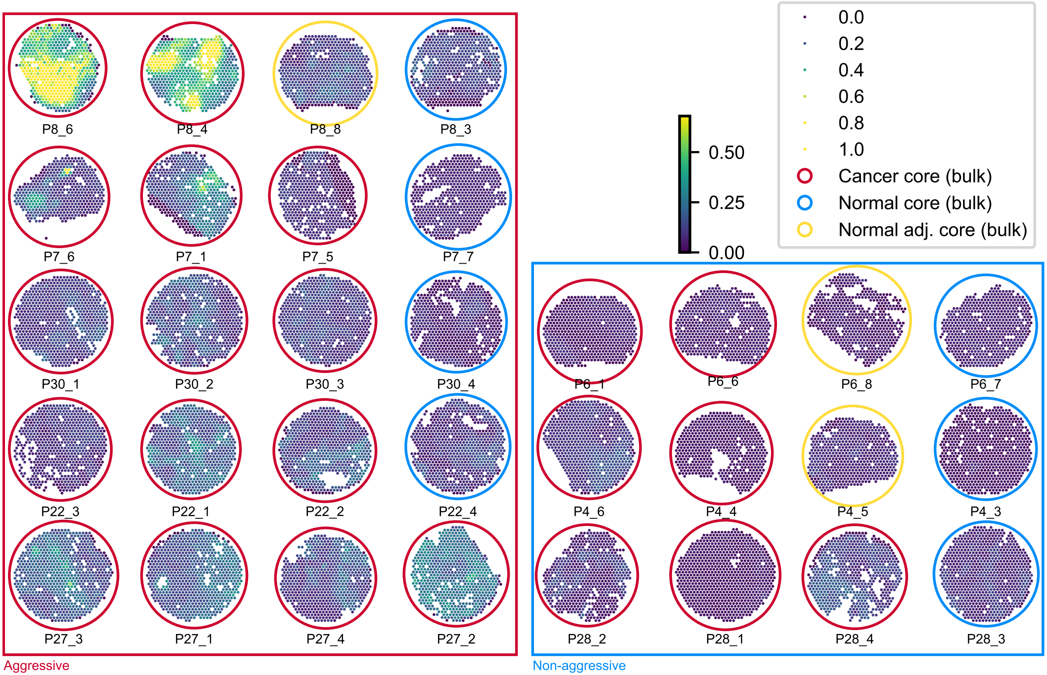

Ion images of NAA (m/z 174.04 +/- 174.04 Da/ +/- 400 ppm, root mean square (RMS) normalized highest intensity) detected in negative mode using MALDI-TOF MSI were registered to Spatial transcriptomics (ST) spots using our Multi-Omics Imaging Integration Toolset (MIIT). Spots are colored using the virids color map representing intensities from 0 to 0.68 covering the 0 to 0.99 quantile of the data as indicated by the color bar. Values above this are all respresented by the color of the 0.99 quantile value 0.68.

Multi-Omics Imaging Integration Toolset (MIIT) registered MSI data of Zinc

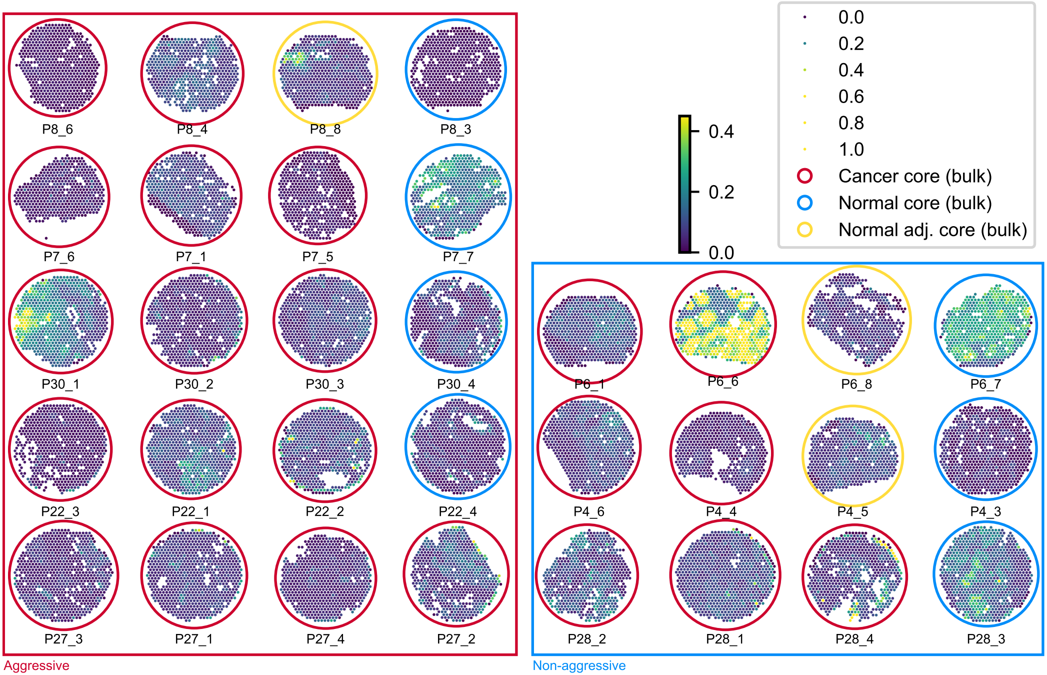

Ion images of Zinc (m/z 174.83 +/- 174.83 Da/ +/- 400 ppm, root mean square (RMS) normalized highest intensity) detected in negative mode using MALDI-TOF MSI were registered to Spatial transcriptomics (ST) spots using our Multi-Omics Imaging Integration Toolset (MIIT). Spots are colored using the virids color map representing intensities from 0 to 0.45 covering the 0 to 0.99 quantile of the data as indicated by the color bar. Values above this are all respresented by the color of the 0.99 quantile value 0.45.

Multi-Omics Imaging Integration Toolset (MIIT) registered MSI data of Citrate

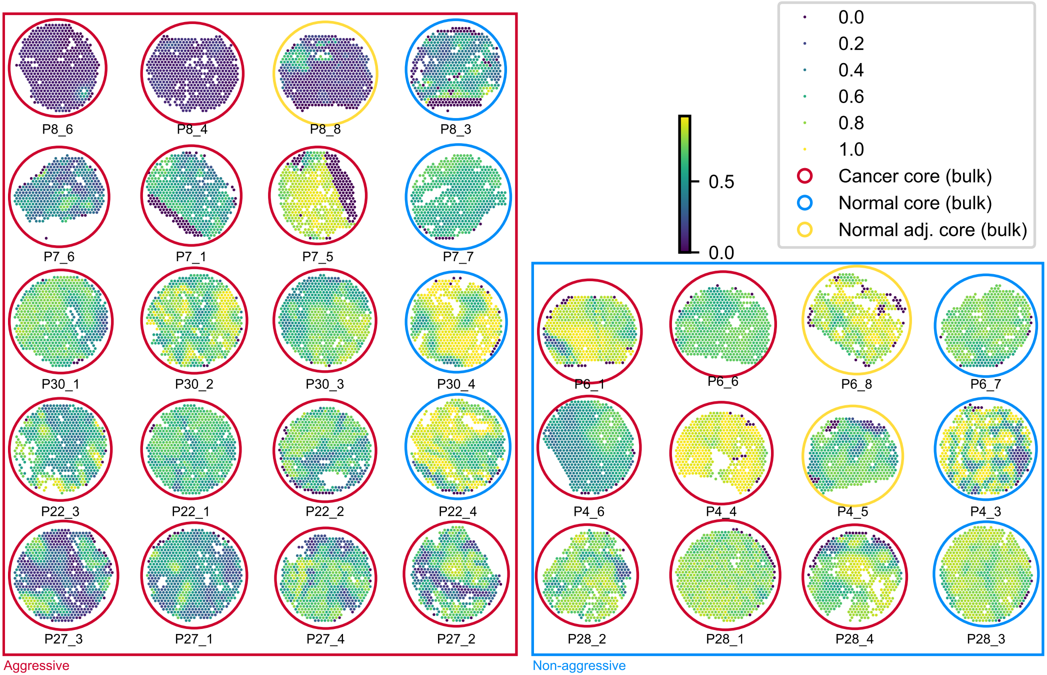

Ion images of Citrate (m/z 191.02 +/- 191.02 Da/ +/- 400 ppm, root mean square (RMS) normalized highest intensity) detected in negative mode using MALDI-TOF MSI were registered to Spatial transcriptomics (ST) spots using our Multi-Omics Imaging Integration Toolset (MIIT). Spots are colored using the virids color map representing intensities from 0 to 0.96 covering the 0 to 0.99 quantile of the data as indicated by the color bar. Values above this are all respresented by the color of the 0.99 quantile value 0.96.

Multi-Omics Imaging Integration Toolset (MIIT) registered MSI data of Glucose

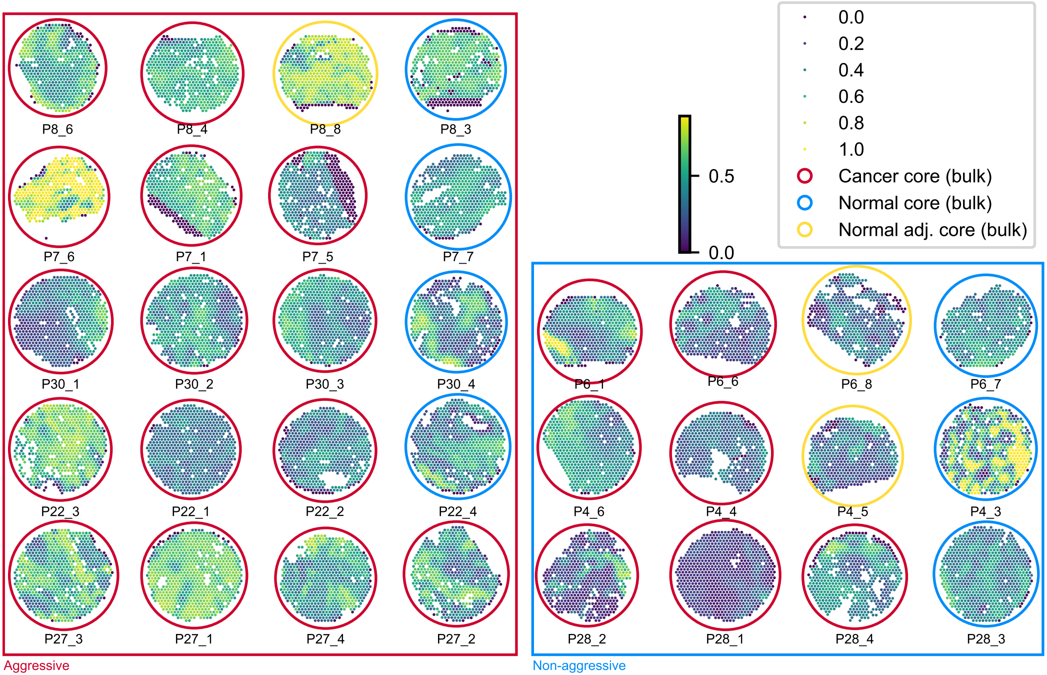

Ion images of Glucose (m/z 215.03 +/- 215.03 Da/ +/- 400 ppm, root mean square (RMS) normalized highest intensity) detected in negative mode using MALDI-TOF MSI were registered to Spatial transcriptomics (ST) spots using our Multi-Omics Imaging Integration Toolset (MIIT). Spots are colored using the virids color map representing intensities from 0 to 0.89 covering the 0 to 0.99 quantile of the data as indicated by the color bar. Values above this are all respresented by the color of the 0.99 quantile value 0.89.

Multi-Omics Imaging Integration Toolset (MIIT) registered MSI data of GSH

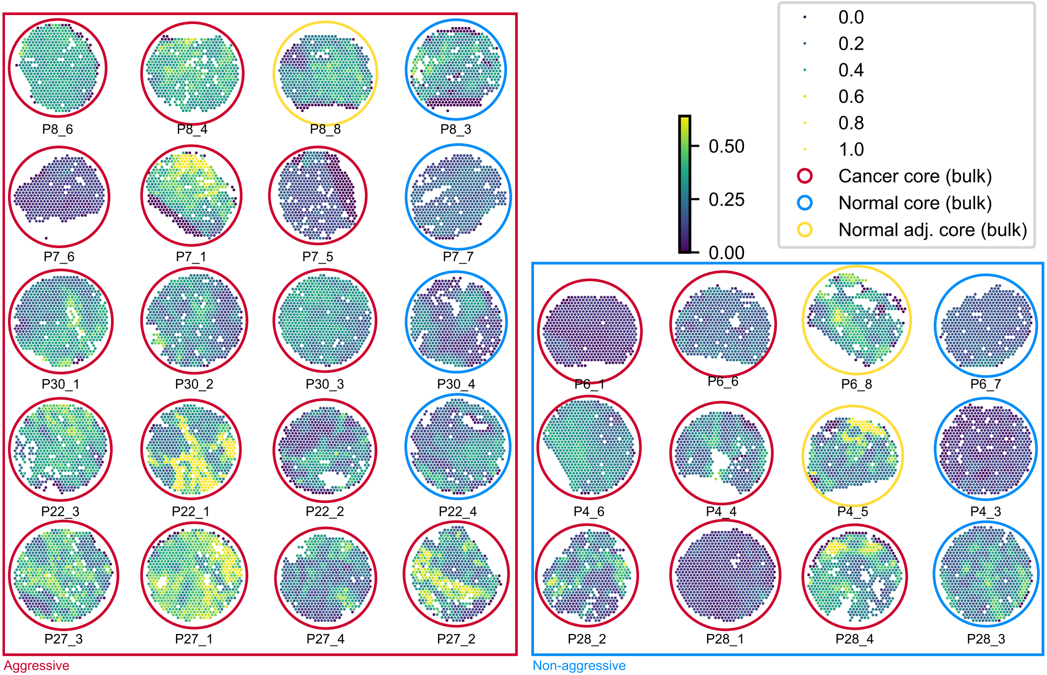

Ion images of GSH (m/z 306.07 +/- 306.068 Da/ +/- 400 ppm, root mean square (RMS) normalized highest intensity) detected in negative mode using MALDI-TOF MSI were registered to Spatial transcriptomics (ST) spots using our Multi-Omics Imaging Integration Toolset (MIIT). Spots are colored using the virids color map representing intensities from 0 to 0.64 covering the 0 to 0.99 quantile of the data as indicated by the color bar. Values above this are all respresented by the color of the 0.99 quantile value 0.64.

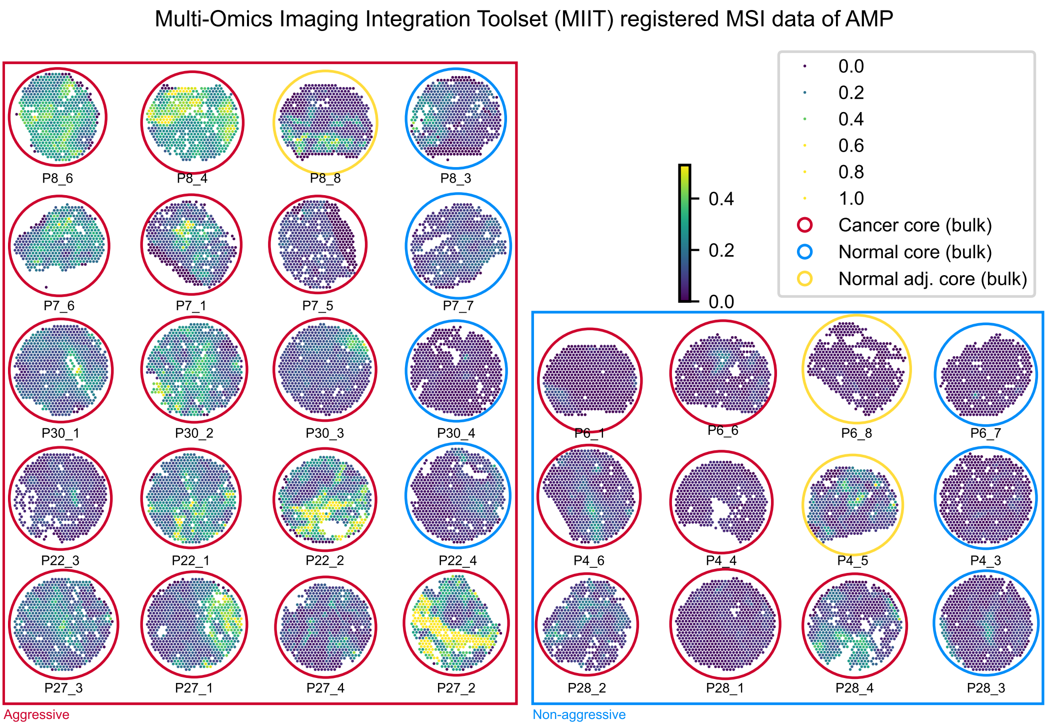

Ion images of AMP (m/z 346.05 +/- 346.05 Da/ +/- 400 ppm, root mean square (RMS) normalized highest intensity) detected in negative mode using MALDI-TOF MSI were registered to Spatial transcriptomics (ST) spots using our Multi-Omics Imaging Integration Toolset (MIIT). Spots are colored using the virids color map representing intensities from 0 to 0.53 covering the 0 to 0.99 quantile of the data as indicated by the color bar. Values above this are all respresented by the color of the 0.99 quantile value 0.53.

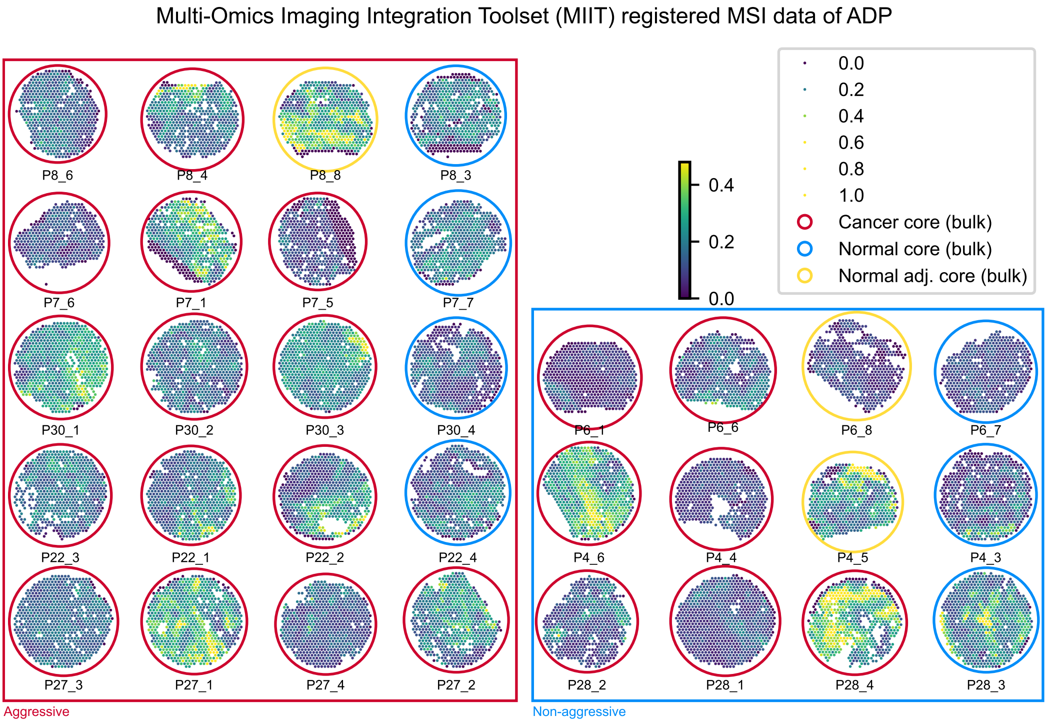

Ion images of ADP (m/z 426.01 +/- 426.01 Da/ +/- 400 ppm, root mean square (RMS) normalized highest intensity) detected in negative mode using MALDI-TOF MSI were registered to Spatial transcriptomics (ST) spots using our Multi-Omics Imaging Integration Toolset (MIIT). Spots are colored using the virids color map representing intensities from 0 to 0.48 covering the 0 to 0.99 quantile of the data as indicated by the color bar. Values above this are all respresented by the color of the 0.99 quantile value 0.48.

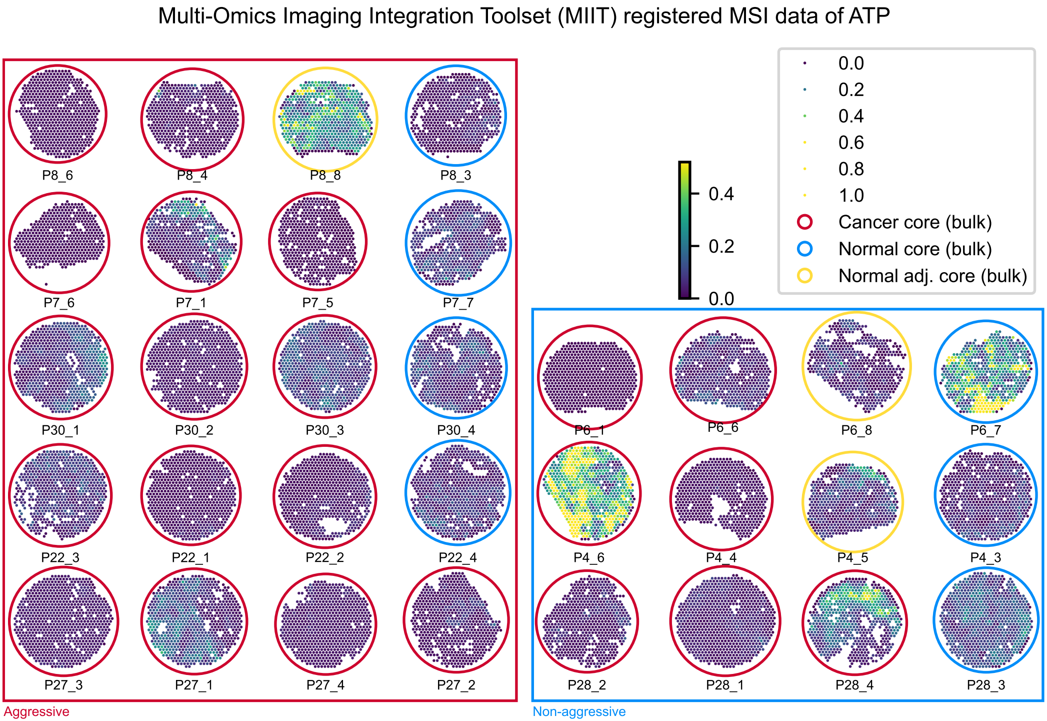

Ion images of ATP (m/z 505.97 +/- 505.97 Da/ +/- 400 ppm, root mean square (RMS) normalized highest intensity) detected in negative mode using MALDI-TOF MSI were registered to Spatial transcriptomics (ST) spots using our Multi-Omics Imaging Integration Toolset (MIIT). Spots are colored using the virids color map representing intensities from 0 to 0.52 covering the 0 to 0.99 quantile of the data as indicated by the color bar. Values above this are all respresented by the color of the 0.99 quantile value 0.52.

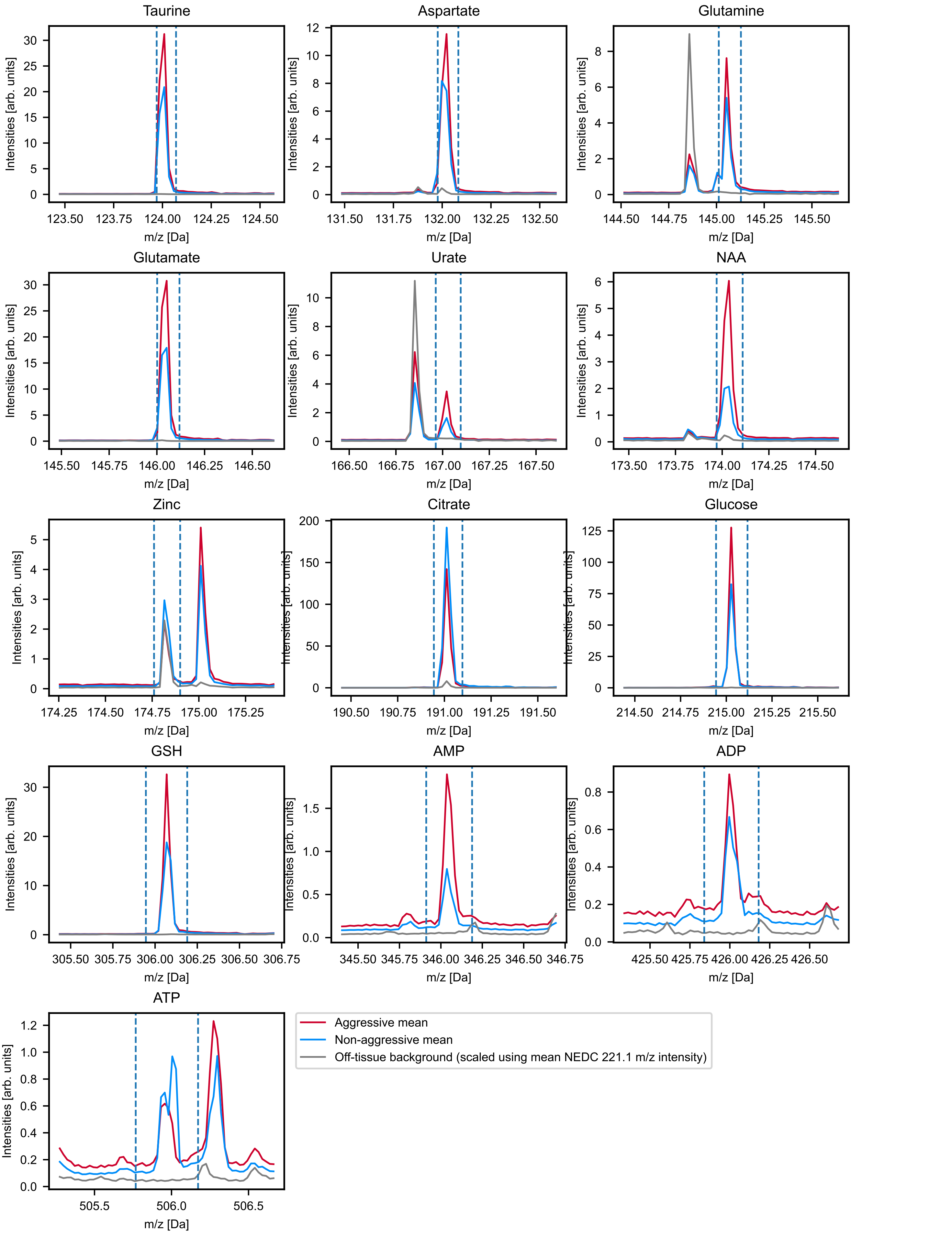

Mean root mean square normalized spectra of all MSI samples grouped by relapse status and off-tissue background spectra collected on each slide. Shown are the regions of the used metabolites in individual sub-plots. The +/- 400 ppm range used for determine the intensity for the ion images is indicated by the dashed vertical lines. To adjust for the pronounced background signals in the off-tissue regions, its mean spectrum was additionally normalized to the mean NEDC (221.1 m/z) on-tissue intensity. The peak-split observed for the mean spectra of ATP were due to a slight mass shift in one of the imaging batches.
